# Small Molecules Targeting DNA Polymerase Theta (POLθ) as Promising Synthetic Lethal Agents for Precision Cancer Therapy

**DOI:** 10.1021/acs.jmedchem.2c02101

**Published:** 2023-05-03

**Authors:** Maria Chiara Pismataro, Andrea Astolfi, Maria Letizia Barreca, Martina Pacetti, Silvia Schenone, Tiziano Bandiera, Anna Carbone, Serena Massari

**Affiliations:** † Department of Pharmaceutical Sciences, University of Perugia, Via del Liceo 1, 06123 Perugia, Italy; ‡ Department of Pharmacy, University of Genoa, Viale Benedetto XV 3, 16132 Genoa, Italy; § D3 PharmaChemistry, Istituto Italiano di Tecnologia, Via Morego 30, 16163 Genova, Italy

## Abstract

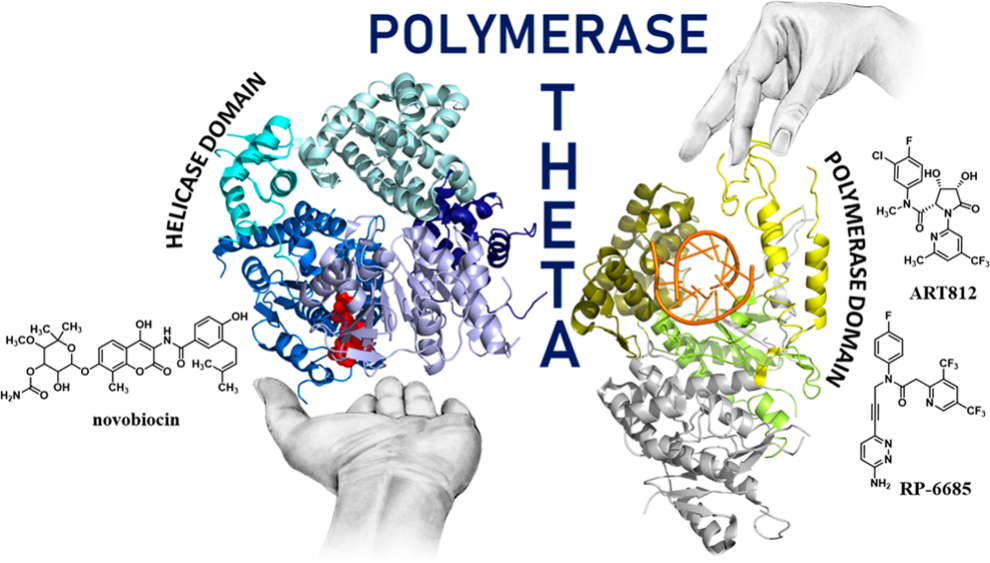

Synthetic lethality (SL) is an innovative strategy in targeted anticancer therapy that exploits tumor genetic vulnerabilities. This topic has come to the forefront in recent years, as witnessed by the increased number of publications since 2007. The first proof of concept for the effectiveness of SL was provided by the approval of poly(ADP-ribose)polymerase inhibitors, which exploit a SL interaction in BRCA-deficient cells, although their use is limited by resistance. Searching for additional SL interactions involving BRCA mutations, the DNA polymerase theta (POLθ) emerged as an exciting target. This review summarizes, for the first time, the POLθ polymerase and helicase inhibitors reported to date. Compounds are described focusing on chemical structure and biological activity. With the aim to enable further drug discovery efforts in interrogating POLθ as a target, we propose a plausible pharmacophore model for POLθ-pol inhibitors and provide a structural analysis of the known POLθ ligand binding sites.

## 1 Introduction

Precision medicine represents a promising therapeutic approach in which therapies are selected based on the patient genotyping.^1,2^ In cancer, tumor development and progression are often driven by changes in different genes (e.g., mutation, upregulation, or downregulation) that cooperate to ensure a growth advantage to cancer cells. However, certain changes have to be present simultaneously in order to guarantee survival of cancer cells, thereby representing cancer-specific vulnerabilities that can be exploited to selectively target the tumor.^3^ In this context, cellular pathways responsible for the repair of chromosomal double-strand breaks (DSBs) play pivotal roles in cell growth and cancer development.^4,5^ In particular, DNA repair-deficient cancer cells are provided with selective growth advantage, leading to genetic instability and the promotion of tumor evolution, but they often become dependent on backup pathways, which represent a cancer-specific vulnerability that can be targeted to kill tumor cells.^6,7^

Mammalian cells employ four pathways to repair the highly cytotoxic DSBs, thereby maintaining genomic integrity and cellular viability: nonhomologous end-joining (NHEJ), homologous recombination (HR), single-strand annealing (SSA), and alternative end joining (alt-EJ) or microhomology-mediated end-joining (MMEJ).^8^ Two genetically distinct mechanisms of MMEJ exist, of which the polymerase theta-mediated end-joining (TMEJ) is the main one.^9^

NHEJ directly ligates broken DNA ends and preferentially repairs unresected DSB ends, whereas HR, SSA, and TMEJ require 5′ to 3′ nucleolytic resection of broken ends to generate ends with 3′ single-stranded DNA (ssDNA) overhangs. The two major pathways, NHEJ and HR, are responsible for repairing the majority of DSBs.

NHEJ is the pathway preferentially adopted by the mammalian cells to resolve DSBs and is active throughout the cell cycle.^10^ It promotes direct ligation of DNA ends through synapsis, end processing, and ligation processes. The first component of the NHEJ pathway recruited at the DSB is the Ku70–Ku80 (Ku) dimer,^11^ which binds to the two DNA break ends and protects them from end-resection. Ku dimer acts as a “tool belt”, interacting and stabilizing other NHEJ proteins, including DNA-dependent protein kinase catalytic subunit (DNA-PKcs) and DNA ligase IV.^11,12^

HR is a conservative error-free DNA repair process limited to the S- and G2 phases.^13^ HR is initiated by MRN complex (MRE11, RAD50, and NBS1), which generates the 3′ single-stranded DNA^14^ and requires BRCA1 first and then BRCA2 to recruit RAD51.^15^ RAD51 is a recombinase capable of performing the essential steps of the HR repair pathway, including strand invasion, homology search on the sister chromatid and strand exchange.^16^ Because the repair of DSB occurs in the presence of homologous sister chromatid as a template, HR is considered accurate and any missing genetic information lost in the DSB or during end processing is recovered.

A cell can use either NHEJ or HR pathways, but the exact reason why a cell opts for one pathway over another is unclear, even because the choice of repair mechanism is continuously adjusted throughout the cell cycle.^17^ However, several factors may influence this choice including the break type, the cell cycle phase in which the damage occurred, and the local chromatin environment.^18,19^ Furthermore, the expression, activity, and availability of repair complex components play a key role in the balance between the repair pathways. In particular, post-translational modifications mediated by cyclin-dependent kinases regulate DNA repair according to the cell-cycle stage.^20,21^

TMEJ is an error-prone repair mechanism able to join 5′-resected substrates by locating and pairing microhomologies present in 3′-overhanging single-stranded tails. It involves poly(ADP-ribose) polymerase (PARP) 1, DNA ligase III, DNA polymerase theta (POLθ), and traditional DNA resection factors.^22−26^ The initial end resection is performed by MRN complex, a step shared with the HR pathway.^27^ Then, TMEJ proceeds through the annealing of exposed microhomologous sequences terminal or internal to the broken ends, the fill-in synthesis of the flanking single-stranded regions, the cleavage of the extraneous 3′-DNA, and the final ligation step where the DNA ends are covalently bound to each other.^28^ Readers are directed to the paper of Pomeranz and co-workers for a comprehensive description on the TMEJ.^29^ TMEJ serves as an essential backup pathway to repair DSBs when NHEJ or HR are compromised, although it was demonstrated that, in some contexts, TMEJ is preferred even when HR and NHEJ are efficient.^30,31^

Many cancers are defective in canonical DNA repair pathways and compensated for through activation of backup pathways for survival, the inhibition of which kills only cancer cells, with fewer side effects in noncancerous cells.^19^ This concept is at the basis of synthetic lethality (SL), a revolutionary approach for the development of new antitumor agents for precision oncology.^32−35^

SL, first discovered in the early 1920s in fruit flies (*Drosophila melanogaster*) by Calvin Bridge^36^ and then observed in 1946 by Theodore Dobzhansky in *Drosophila pseudoobscura*,^37^ describes the relationship between two genes in which alteration (e.g., mutation, overexpression, inhibition) of one gene is tolerated, whereas combined dysfunction of both genes causes cell death.^38^ In 1997, Hartwell and colleagues exploited the concept of SL to develop anticancer-selective agents.^39^ Specifically, they showed that pharmacological inhibition of a pathway that is synthetically lethal with a cancer-specific alteration is lethal for tumor cells, sparing nonmalignant cells.^40^

Evidence of the effectiveness of this approach was provided by the approval of PARP inhibitors (PARPi), which exploit a SL interaction in HR deficient and particularly *BRCA*-deficient cells. These cells are unable to repair the collapsed replication forks and double-strand breaks, which result from PARP inhibition and trapping on DNA.^41,42^ HR defective *BRCA1/2* mutant tumors fail to repair PARP inhibition-induced damage, while normal cells that have an intact HR pathway are largely unaffected by PARPi treatments.^43^

The clinical success of PARPi provided a proof-of-principle of the SL approach.^44^ To date, four PARP1/2 inhibitors, **olaparib**,^45−49^**rucaparib**,^50,51^**niraparib**,^52^ and **talazoparib**,^53^ have received marketing authorization in the United States and/or Europe. Despite the encouraging therapeutic benefits of PARPi, these agents show some critical issues, such as suboptimal clinical efficacy, ineffectiveness in certain DNA-repair deficient cancers, and development of acquired and innate resistance.^54,55^ The major resistance mechanism observed in preclinical and clinical models was related to the reversion mutation in *BRCA2*, which allows the correct encoding of functional proteins, thus restoring the HR pathway. Several additional resistance mechanisms have been investigated in preclinical *in vitro* and *in vivo* models, such as a nonreversion-based mechanism that restores HR pathway independently from *BRCA* genes (e.g., loss of 53BP1), PARP1 mutation, and reduction of the drug concentration in cells by upregulation of efflux pumps.^56^

For these reasons, there is an urgent need and opportunity to identify other synthetic lethal partners involved in DNA repair pathways for the development of novel tailored therapies for *BRCA*-deficient tumors. In addition to the interaction between PARP inhibition and mutations in *BRCA1*/2, numerous other synthetic lethal interactions have been discovered in cancer.^57^ In recent years, a number of small molecules targeting DNA damage response have been reported, including inhibitors of DNA-dependent protein kinase (DNA-PK), ataxia-telangiectasia and Rad3 related (ATR), ataxia-telangiectasia mutated (ATM), CHK1, WEE1, and POLθ, some of which are currently being tested in clinical trials.^58−60^

Among these synthetic lethal targets, the low-fidelity POLθ (encoded by *PolQ* gene) emerged as particularly promising for the treatment of *BRCA1*-deficient cancers.^61^ The first POLθ cellular role was identified in *Drosophila melanogaster* through the analysis of mus308 mutants hypersensitive to DNA cross-linking agents.^62,63^ Subsequently, POLθ activity was associated with the backup DNA error-prone repair pathway TMEJ,^64^ in which it promotes end-joining of 3′ssDNA overhangs at DSB site.^29^ In particular, after synapses formation, POLθ promotes first the annealing of both terminal and internal sequence microhomology (2–6 base pairs), and then the fill-in synthesis to extend the annealed overhangs. During terminal transferase activity, POLθ oscillates between three different modes of nucleotide insertion: templated in cis (snap-back), templated in trans, and nontemplated.^65^

Recently, the end-trimming activity of POLθ has been reported, which depends on dNTP-dependent endonuclease and allows processing of DNA ends prior to DNA synthesis.^66^

**Figure 1:**
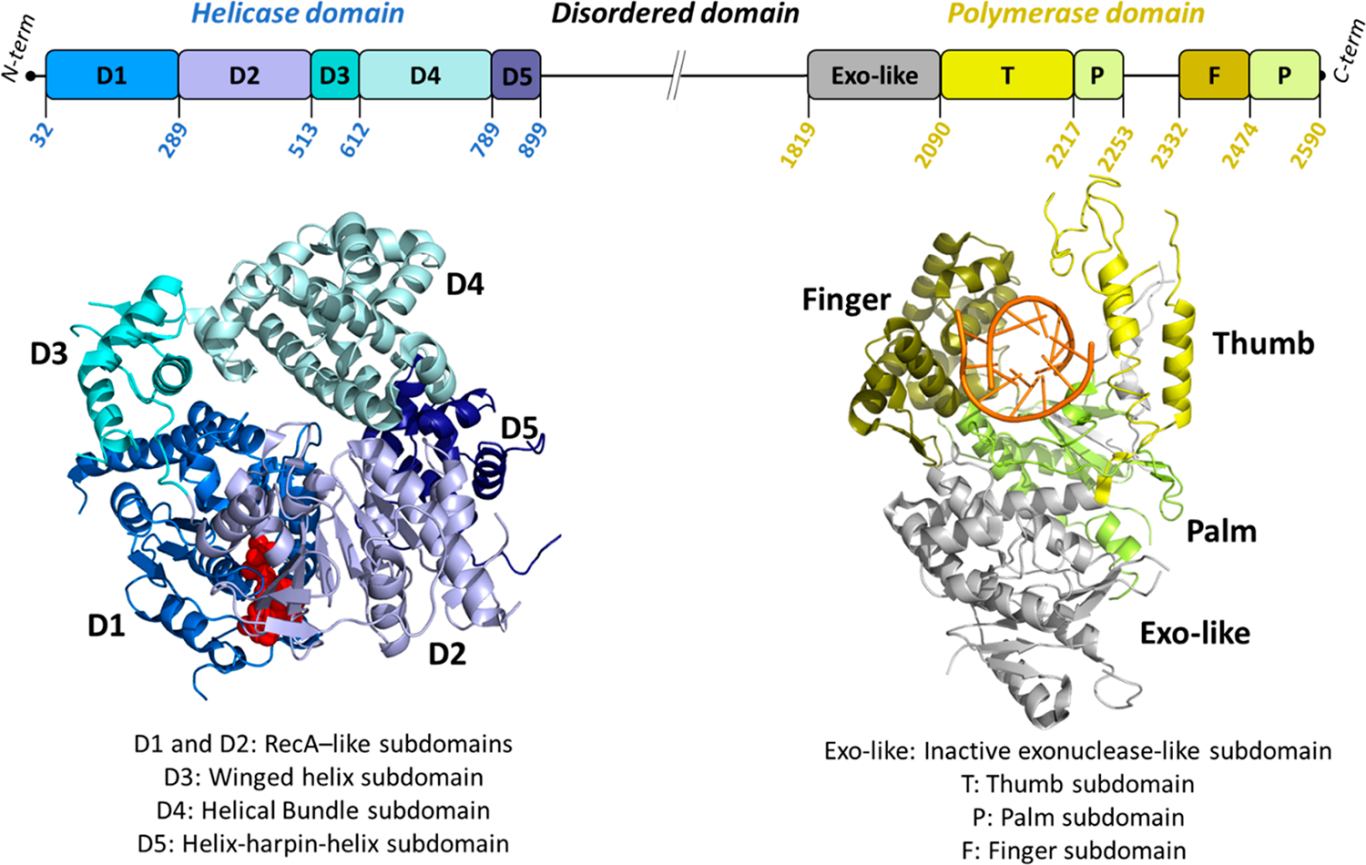
Schematic organization of the full-length POLθ domains and their spatial arrangement on POLθ structure. The cartoon representations (POLθ-hel domain, PDB 5AGA;^69^ POLθ-pol domain, PDB 6XBU(70)) are color-coded according to the domain organization. Red CPK sphere: adenosine 5′-(β,γ-imino) triphosphate (AMP-PNP); orange cartoon, DNA:RNA duplex.

POLθ belongs to the A-family polymerase and is a large size (2590 residues in humans) multifunctional protein containing a superfamily II N-terminal conserved helicase-like domain (referred to as POLθ-hel, residues 32–899) and a C-terminal conserved DNA polymerase domain (referred to as POLθ-pol, residues 1819–2590), linked by an unstructured central region (Figure 1). Both POLθ-hel and POLθ-pol domains are crucial for TMEJ activity, while the central portion probably has a regulatory role.^67,68^

POLθ is the only eukaryotic polymerase with a helicase domain. POLθ-hel is involved in the ATPase dependent displacement of replication protein A (RPA) from DNA to promote TMEJ at the expense of HR. These helicase activities favor the annealing of microhomologies, thus allowing the extension of the annealed overhangs by POLθ-pol.^69,71−73^ Moreover, the ability of POLθ-hel to unwind, in both ATP-dependent and -independent manner, different types of DNA substrates with 3′-5′ polarity (replication forks, blunt-ended DNA, and 3′ or 5′ overhangs) and RNA:DNA hybrids has been recently demonstrated by biochemical assays.^74^

In addition to its polymerase activity in TMEJ, POLθ-pol is involved in translesion synthesis incorporating nucleotides opposite an abasic site or thymine glycol lesions,^75^ in base excision repair (BER) of DNA damage through its 5′-deoxyribose phosphate lyase functions,^76^ and in replication fork repair.^77^ Moreover, POLθ also functions during the first steps of DNA replication, influencing the timing of replication initiation,^78^ and it is implicated in hypermutation during antibody maturation.^79,80^

Interestingly, POLθ is overexpressed in several human cancers (e.g., breast, ovarian, lung, gastric, and colorectal), whereas in most normal cells it is either expressed at a low level or completely absent, making this enzyme an ideal cancer-selective target.^81−84^ The depletion of POLθ by siRNA and shRNA was demonstrated to be useful: (i) in *BRCA1*-deficient cells in combination with PARPi treatment, leading to synergistic antitumor effects and a possible delay of PARPi resistance,^61^ (ii) to prevent the onset of PARPi resistance, as well as that of *cis*-platinum, due to TMEJ-dependent functional reversion of *BRCA2* mutations,^56,85^ (iii) to improve radiosensitization of different HR-proficient^86−88^ as well as p53-mutated^89^ tumor cells, and (iv) to sensitize cancer cells to other chemotherapeutic agents, such as topoisomerases I and II inhibitors.^77^ In addition, POLθ showed synthetically lethal interactions with other DNA repair tumor suppressor genes, such as *FANCD2*, *RAD51C*, and *PALB2* involved in the HR pathway, *RAD52* mediating SSA/BIR, *ATM* and *ATR* related with HR functionalities, and *Ku70* and *TP53BP1* controlling NHEJ.^90,91^

All these findings suggest that POLθ activity inactivation would provide a novel targeted therapeutic strategy in a variety of tumor context, including HR- and NHEJ-deficient cancers.^90,92,93^ However, POLθ is still largely unexplored as drug target and the potential to exploit POLθ/HR-genes synthetic lethal interaction using small molecule POLθ inhibitors is only at an early stage. The first potent and selective inhibitors of POLθ DNA polymerase and ATPase activities have been recently reported by several pharmaceutical companies, one of which just entered clinical trials for the treatment of advanced or metastatic solid tumors.

The aim of this review is to describe the current status of the POLθ inhibitor research field. An overview of all of the POLθ small molecule inhibitors reported so far and their use in the treatment of HR-deficient cancer and Shieldin deficiency associated cancer is provided for the first time. Compounds are classified according to their mechanism of action, i.e., POLθ-pol or POLθ-hel inhibitors, trying to highlight structural similarities and differences, as well as the approaches used for their identification and optimization. Furthermore, the biochemical data and the number of compounds described as POLθ-pol allosteric inhibitors allowed us to define their common chemical features and propose a 3D pharmacophore model amenable for ligand-based studies. Finally, a brief analysis of the available POLθ crystallographic structures is provided, focusing on known inhibitor binding sites.

## 2 Small Molecule POLθ Inhibitors

The identification of POLθ inhibitors is a research field that has only recently attracted interest from the scientific community and, particularly, from pharmaceutical companies. Most of the compounds described to date have been reported in 16 PCT Patent Applications (PAs) filed between 2016 and 2021, while the only five original papers reporting POLθ inhibitors were published the first one in 2016,^94^ the next two in 2021, curiously on the same day,^95,96^ and the last two in 2022.^97,98^ The latter, made public while we were about to finish this review, are noteworthy because they report, for the first time ever, medicinal chemistry studies on POLθ inhibitors.

The PAs and original papers with relative details are summarized in Table 1. Among the 16 PAs, 14 reported small molecules as POLθ inhibitors, while 2 reported their use for the treatment of HR-deficient tumors. Moreover, 10 and 6 PAs focused on inhibitors of POLθ-pol and POLθ-hel activity, respectively. To the best of our knowledge, the first PA reporting POLθ inhibitors was filed by Temple University of the Commonwealth system of Higher Education (Temple University) and is the unique reporting nucleotide analogues as polymerase activity inhibitors.^99^ All the other PAs were filed by (i) Artios Pharma Limited (Artios), a leading independent DDR company, which focuses on POLθ-pol inhibitors,^100−104^ (ii) Dana-Farber Cancer Institute, Inc. (Dana-Farber), specialized on precision oncology therapies, which focused on POLθ-hel inhibitors,^105−108^ and (iii) Ideaya Biosciences, Inc. (Ideaya), a SL-focused precision medicine oncology company that reported both classes of compounds.^109−114^ Of note, one of the most recent original papers,^97^ which reported the development of POLθ-pol inhibitors, was also published by a pharmaceutical company, Repare Therapeutics, a clinical-stage precision oncology company focused on SL. A brief description of each PA and original manuscript is given below, dividing them according to the mechanism of action of the described compounds, i.e., polymerase and helicase inhibitors.

**Table 1:** Summary of the PAs and Original Papers Reporting POLθ Inhibitors and/or Their Use for the Treatment of HR-Deficient Tumors (Reported up to March 31, 2023)

PA no./DOI	applicant	PCT filing date and publication date (dd.mm.yyyy)	figure	POLθ domain inhibited	ref
DOI: 10.1093/nar/gkw721	Temple University of the Commonwealth system of Higher Education	06.08.2016	2	polymerase	(94)
WO2018/035410A1	Temple University of the Commonwealth system of Higher Education	18.08.2017	2	polymerase	(99)
		22.02.2018			
WO2020/160213A1	Ideaya Biosciences, Inc.	31.01.2020	3a	polymerase	(109)
		06.08.2020			
WO2020/160134A1	Ideaya Biosciences, Inc.	29.01.2020	3b	polymerase	(110)
		06.08.2020			
WO2022/026548A1	Ideaya Biosciences, Inc.	28.07.2021	3c	polymerase	(111)
		03.02.2022			
WO2022/026565A1	Ideaya Biosciences, Inc.	28.07.2021	3d	polymerase	(112)
		03.02.2022			
WO2021/028643A1	Artios Pharma Limited	09.08.2019	3e	polymerase	(100)
		18.02.2021			
WO2021/123785A1	Artios Pharma Limited	17.12.2020	3f	polymerase	(101)
		24.06.2021			
WO2021/028644A1	Artios Pharma Limited	09.08.2019	-	polymerase	(102)
		18.02.2021			
WO2020/030924A1	Artios Pharma Limited	09.08.2019	4a	polymerase	(103)
		13.02.2020			
WO2020/030925A1	Artios Pharma Limited	09.08.2019	4b	polymerase	(104)
		13.02.2020			
DOI: 10.1038/s41467-021-23463-8	Artios Pharma Limited	17.06.2021	-	polymerase	(95)
DOI: 10.1021/acs.jmedchem.2c00998	Repare Therapeutics	20.09.2022	5	polymerase	(97)
DOI: 10.1021/acs.jmedchem.2c01142	Artios Pharma Limited	06.10.2022	6	polymerase	(98)
WO2020/243459A1	Ideaya Biosciences, Inc.	20.05.2020	10a	helicase	(113)
		03.12.2020			
WO2022/118210Al	Ideaya Biosciences, Inc.	01.12.2021	10b	helicase	(114)
		09.06.2022			
WO2017/070198A1	Dana-Farber Cancer Institute, Inc.	19.10.2016	-	helicase	(105)
		27.04.2017			
WO2019/079297A1	Dana-Farber Cancer Institute, Inc.	16.10.2018	11a	helicase	(106)
		25.04.2019			
WO2021/046220A1	Dana-Farber Cancer Institute, Inc.	03.09.2020	11b	helicase	(107)
		11.03.2021			
WO2021/046178A1	Dana-Farber Cancer Institute, Inc.	03.09.2020	11c	helicase	(108)
		11.03.2021			
DOI: 10.1038/s43018-021-00203-x	Dana-Farber Cancer Institute, Inc.	17.06.2021	-	helicase	(96)

### 2.1 POLθ Polymerase Inhibitors

**Figure 2:**
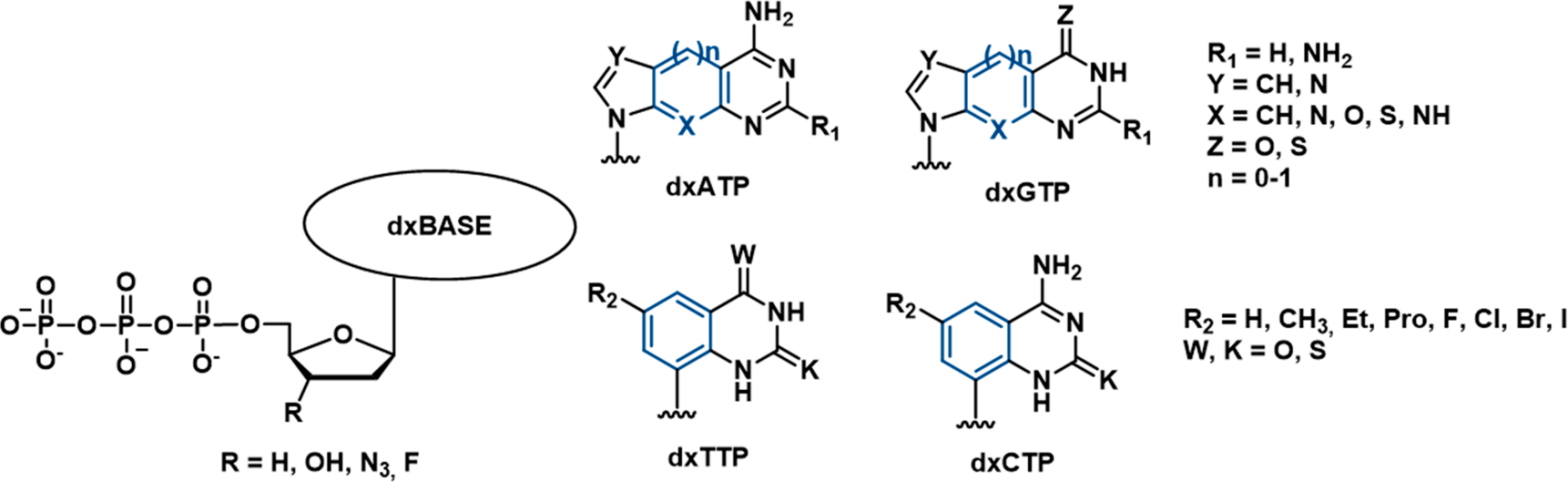
Structure of **xDNA nucleotides** and analogues as POLθ-pol inhibitors reported in PA WO2018/035410Al^99^ by Temple University and in the paper by Kent et al.^94^

To best of our knowledge, the first PA describing POLθ inhibitors was filed by Temple University in 2017 (WO2018/035410A1),^99^ which comprises concepts and compounds described in a previous manuscript, published in 2016.^94^ The PA reports synthetic expanded-size DNA (**xDNA**, Figure 2) **nucleotides** and analogues that were efficiently recognized by POLθ as substrates leading to the inhibition of the POLθ DNA synthesis activity. The expanded-size deoxyribonucleotides were characterized by the presence of an additional four-, five-, or six-membered ring within the original base moiety, which was also decorated with different substituents. The compounds were investigated as expanded-size deoxyribonucleoside monophosphates (dxNMPs) and triphosphates (dxNTPs) and monophosphate prodrugs. The incorporation of expanded-size dxNMPs into DNA by POLθ was determined by evaluating the ability of the purified POLθ-pol domain to perform primer extension in the presence of a single complementary benzo-expanded dxNTP *in vitro*. Benzo-expanded dxNTPs were known to form canonical base pairs and increase the width of double-stranded DNA by 2.4 Å. Moreover, the dxNMPs bases exhibited strong base stacking interactions. All four dxNTPs were efficiently used by POLθ as substrates for primer-template extension. On the other hand, Y-family (Polκ and Polη), X-family (Polβ), B-family (Polδ, Polε Polα), and A-family (Polγ) polymerases failed to incorporate dxNTPs into DNA or showed a significant reduced ability to integrate xDNA analogues compared to POLθ. Moreover, consecutive or closely spaced incorporation of two dxNMPs strongly inhibited the DNA synthesis activity of POLθ, even in the presence of all four original nucleotides, presumably due to distortion of the polymerase active site that could prevent proper positioning of the next incoming nucleotide or suppress forward translocation of the enzyme.

**Figure 3:**
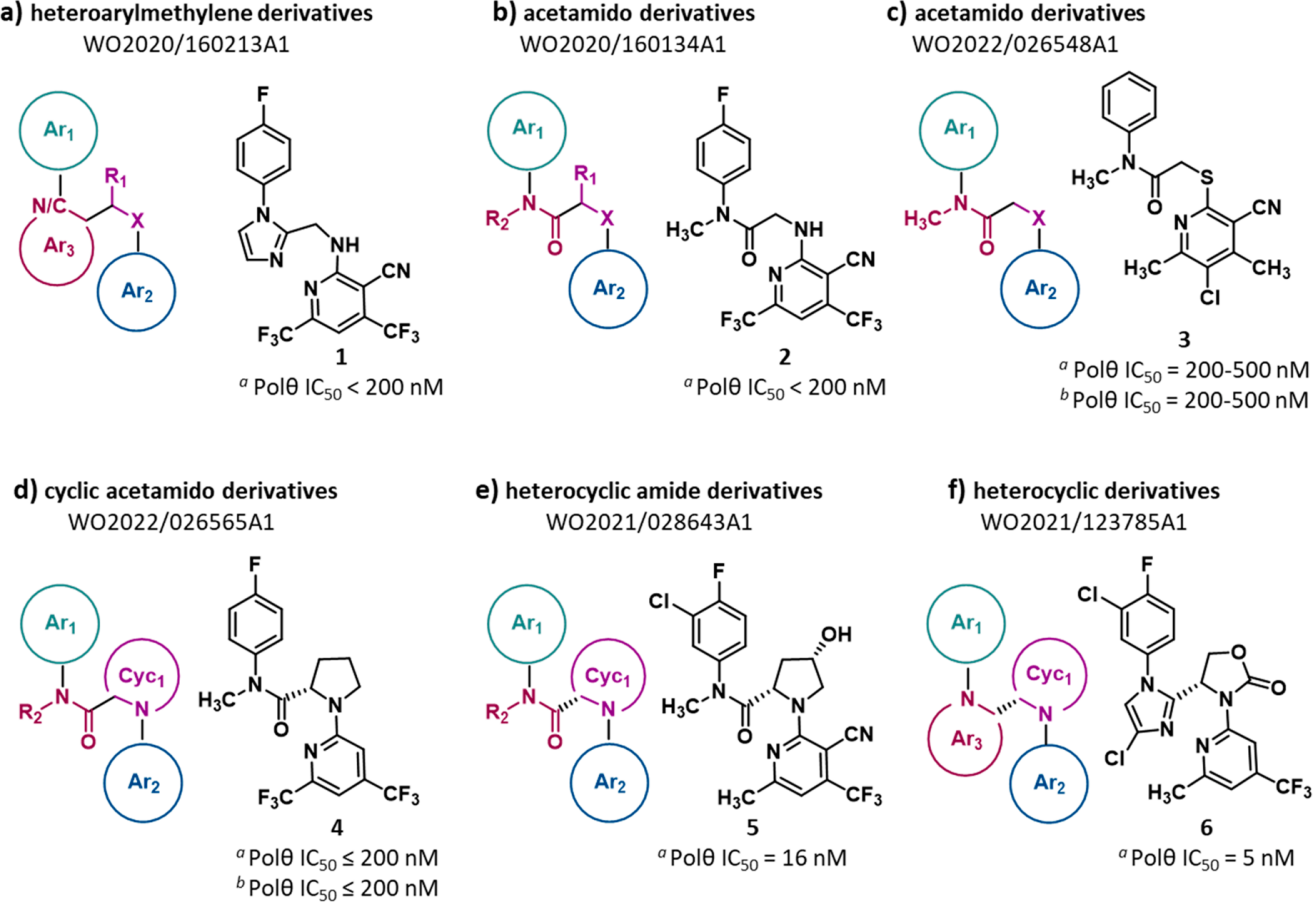
Structure of POLθ-pol inhibitors reported in PAs filed by Ideaya^109−112^ (**3a**–**d**) and Artios^100,101^ (**3e** and **3f**). The IC_50_ value represents the compound concentration that reduces by 50% the POLθ-pol activity as measured by ^(*a*)^PEA and ^(*b*)^PPi assay.

In 2020, Ideaya filed two PAs disclosing POLθ-pol domain inhibitors. PA WO2020/160213A1^109^ reports 27 **heteroarylmethylene derivatives** (exemplified by compound **1**, Figure 3a, and SI, Figure S1), while WO2020/160134A1^110^ discloses 61 strictly related **acetamido derivatives** (exemplified by compound **2**, Figure 3b, and SI, Figure S2). The two series shared two (hetero)aromatic portions, Ar_1_ and Ar_2_, which, in the majority of compounds, were separated by four atoms, i.e., N/C–C–C–X, although in heteroarylmethylene derivatives, the N/C–C atoms were part of a heteroaromatic ring (Ar_3_, Figure 3a), while in acetamido derivatives, N–C was part of a tertiary amide. In the linker, most compounds had a nitrogen atom as heteroatom and an unsubstituted methylene unit. The ability of all the compounds to inhibit POLθ-pol activity was determined using a fluorescence-based primer extension assay (PEA) using POLθ-pol domain (residues 1819–2590). No individual IC_50_ values (compound concentration that reduces by 50% the POLθ-pol activity) were reported for the compounds, whose activity spans from 10 μM to <200 nM. Depending on the IC_50_, each compound was assigned to one of four groups (IC_50_ = 10 μM to 1 μM, = 1 μM to 500 nM, = 500 nM to 200 nM, and <200 nM). Among the reported^27^ heteroarylmethylene derivatives, 19 showed an IC_50_ < 200 nM, while only 4 compounds displayed an IC_50_ = 1–10 μM. Out of the 61 described acetamido derivatives, 41 showed an IC_50_ < 200 nM, while no compounds displayed an IC_50_ > 1 μM. No activity in cellular assays has been reported for any compound, and no follow-up papers have been published at this time.

In 2021, Ideaya filed two additional PAs disclosing inhibitors of POLθ-pol domain. PA WO2022/026548Al^111^ reports 16 additional **acetamido derivatives** (exemplified by compound **3**, Figure 3c, and SI, Figure S3), in which the molecule backbone, i.e., the Ar_1_ and Ar_2_ groups linked by a N–C(O)–C–X belt, was maintained (the heteroatom within the linker was frequently a sulfur atom). PA WO2022/026565Al^112^ discloses 31 **cyclic acetamido derivatives** (exemplified by compound **4**, Figure 3d, and SI, Figure S4), in which the C–X within the N/C–C–C–X linker was part of a nitrogen-based heterocycle. Notably, in the last series, POLθ activity was dependent on the stereochemistry at the C-2 carbon of the pyrrolidine ring. The (*S*)-stereochemistry, as in compound **4** (IC_50_ < 200 nM), is associated with higher potency, whereas the (*R*)-isomer of compound **4** shows an IC_50_ in the 1–10 μM range. Assessment of compounds’ activity was performed using PEA, as for analogues described above, with 5 acetamido derivatives showing an IC_50_ < 200 nM and 3 displaying an IC_50_ in the 1–20 μM range, and 20 cyclic acetamido derivatives showing an IC_50_ < 200 nM and 2 displaying an IC_50_ between 1 and 20 μM. No activity in cell-based assays was reported but, in addition to the PEA, a luminescence-based inorganic pyrophosphate (PPi) assay was used to determine the ability of compound **3** (Figure 3c) and 15 cyclic acetamido derivatives (10 compounds showed an IC_50_ < 200 nM) to inhibit POLθ-pol activity.

Cyclic acetamido derivatives described above are strictly related to those previously disclosed in PA WO2021/028643A1,^100^ the first PA filed by Artios in 2019. In this PA, 245 **heterocyclic amide derivatives** are reported as inhibitors of POLθ-pol domain (exemplified by compound **5**, Figure 3e, and SI, Figure S5). In this series of compounds, many nitrogen-based heterocycles were exploited as Cyc_1_, all characterized by (*S*)-stereochemistry at the carbon atom bearing the amide group. A PicoGreen-based PEA was employed to measure the ability of the compounds to inhibit the POLθ-pol activity *in vitro* by using a full-length protein (amino acids 2–2590) expressed in baculovirus. In contrast to previously reported PAs, individual IC_50_ values are reported for the compounds, ranging from 2 nM to 1.76 μM. The majority of these small molecules were particularly potent, with 203 of the 245 compounds displaying an IC_50_ < 200 nM, of which 179 showing an IC_50_ < 100 nM and 49 compounds an IC_50_ < 10 nM. The medicinal chemistry approach used to identify and optimize some of these compounds has been recently reported in an article,^98^ and one compound (**ART558**) was in depth investigated in another recent publication^95^ (see section 2.1.1.).

In 2020, Artios filed a PA (WO2021/123785A1^101^) disclosing 66 **heterocyclic derivatives** (exemplified by compound **6**, Figure 3f, and SI, Figure S6), which showed the same N/C–C–C–X backbone characterizing all the POLθ-pol inhibitors previously described. Nicely, in these compounds, the N/C–C was part of a nitrogen-based heteroaromatic ring (Ar_3_), analogously to heteroarylmethylene derivatives (Figure 3a), while the C–X was part of a nitrogen-based heterocycle, characterizing cyclic acetamido derivatives (Figure 3d) and heterocyclic amide derivatives (Figure 3e). As described before, the compounds were assayed as POLθ-pol inhibitors in a PEA: their IC_50_ values range from 3 nM to 3.2 μM. Out of the 66 compounds, 42 showed an IC_50_ < 200 nM, of which 23 showing an IC_50_ < 100 nM and 14 derivatives displayed an IC_50_ < 10 nM. No activity in cell-based assays was reported and, to date, no follow-up papers have been published.

**Figure 4:**
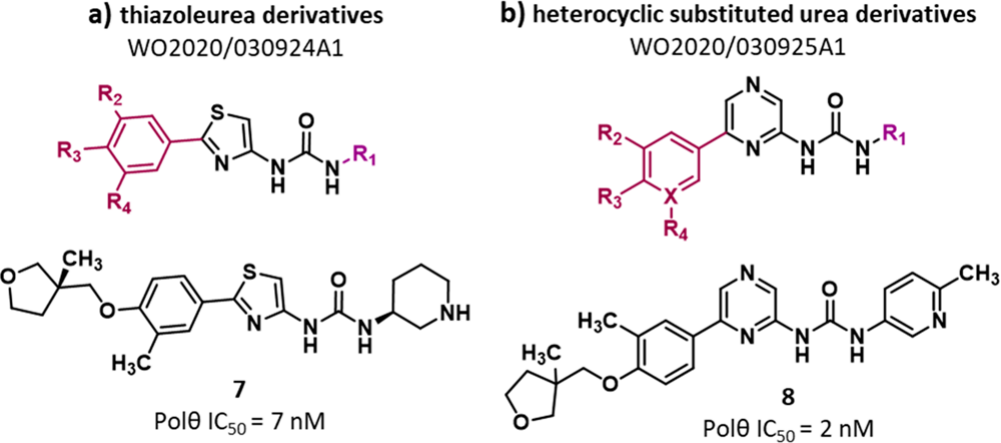
Structure of thiazoleurea derivatives^103^ (a) and heterocyclic substituted urea derivatives^104^ (b) as POLθ-pol inhibitors reported in PAs filed by Artios. The IC_50_ value represents the compound concentration that reduces by 50% the POLθ-pol activity as measured by PEA.

A different chemical structure characterized compounds reported in two PAs filed by Artios in 2019. PA WO2020/030924A1^103^ reports 127 **thiazoleurea derivatives** (exemplified by compound **7**, Figure 4a, and SI, Figure S7) and PA WO2020/030925A1^104^ discloses 215 strictly related **heterocyclic substituted urea derivatives** (exemplified by compound **8**, Figure 4b, and SI, Figure S8). The two series shared a central aromatic core, which was a thiazole in thiazoleurea derivatives and a pyrazine in heterocyclic substituted urea derivative; the core was linked to a substituted (hetero)aromatic ring and an urea moiety further linked to a nitrogen-based aromatic or aliphatic ring. The compounds were evaluated in a PEA, with thiazoleurea derivatives showing IC_50_ values from 7 nM to 1.7 μM. In particular, out of 127 compounds reported, 69 displayed an IC_50_ < 200 nM, of which 47 having an IC_50_ < 100 nM and only compound **7** an IC_50_ < 10 nM. Regarding heterocyclic substituted urea derivatives, a range of IC_50_ values from 2 nM to 9 μM was reported, with 142 compounds showing IC_50_ < 200 nM, of which 96 exhibiting an IC_50_ < 100 nM and 17 compounds an IC_50_ < 10 nM. No activity in cell-based assays was reported and, to date, no follow-up papers are published. Compound **8** was used in two studies, published in 2022, to investigate the basis of genetic interaction between POLθ and BRCA1/2, but no mechanism of action and target engagement studies have been reported.^115,116^

In the first study, Loizou and co-workers investigated if ssDNA gaps, which are considered to be the major lesions that drive cell death in BRCA1/2-deficient cells treated with PARPi or cisplatin, contributes to the genetic interaction between BRCA1/2 and POLθ.^115^ The authors demonstrated that loss of POLθ activity in BRCA1-deficient cells exposes ssDNA, which causes replication stress and deregulation of S-phase progression. In addition, a genome-wide CRISPR-Cas9 knockout screen uncovered two modulators of the functional interaction between BRCA1 and POLθ, the MRN complex and the cyclin-dependent kinase 6 (CDK6), which promote the DNA damage induced by compound **8** treatment through two different cellular processes. Loss of MRN-complex activity suppresses nucleolytic processing of gaps, while CDK6 loss reduces replication stress by diminishing entry into S-phase. In this study, computational docking studies were also performed for compound **8**, suggesting that it binds to an allosteric pocket in the thumb subdomain of POLθ.

Results reported by Loizou and co-workers were similar to those reported by Boulton and co-workers, who demonstrated that POLθ is a potent ssDNA gap filling enzyme.^117^ In particular, cooperation of POLθ-hel activity, which promotes RPA displacement, and POLθ-pol activity, which promotes ssDNA-gap fill-in, impaired the accumulation of ssDNA and postreplicative ssDNA gaps in BRCA1 hypomorphs and BRCA2-depleted cells. Accordingly, accumulation of ssDNA gaps was observed in POLθ-deficient cells upon BRCA1/2 loss or PARPi treatment. In addition, POLθ can promote microhomology-mediated gap skipping by annealing microhomologies present at the 3′ ss-dsDNA junction and within the ssDNA gap, resulting in deletions during gap repair.

In the second study, Costanzo and co-workers revealed a POLθ-dependent genome protection function preventing stalled forks rupture.^116^ In particular, isolated *Xenopus laevis* POLθ was shown to extend the stalled Okazaki fragments by filling of ssDNA through its polymerase domain, leading to suppression of the accumulation of ssDNA fork gaps arising at stalled forks. In agreement, POLθ-pol inhibition by compound **8** resulted in ssDNA gaps accumulation, which predispose to the formation of MRE11-NBS1-CtIP-dependent DSBs in HR-defective cells mainly during the S-phase. These results also raised a question about a possible resistance mechanism to POLθ inhibitors in HR-defective cancer cells with nonlethal cancer mutations in *Mre11-Nbs1-CtIP* genes.

#### 2.1.1 POLθ-pol Inhibitors ART558, ART812, and ART4215

**Figure 5:**
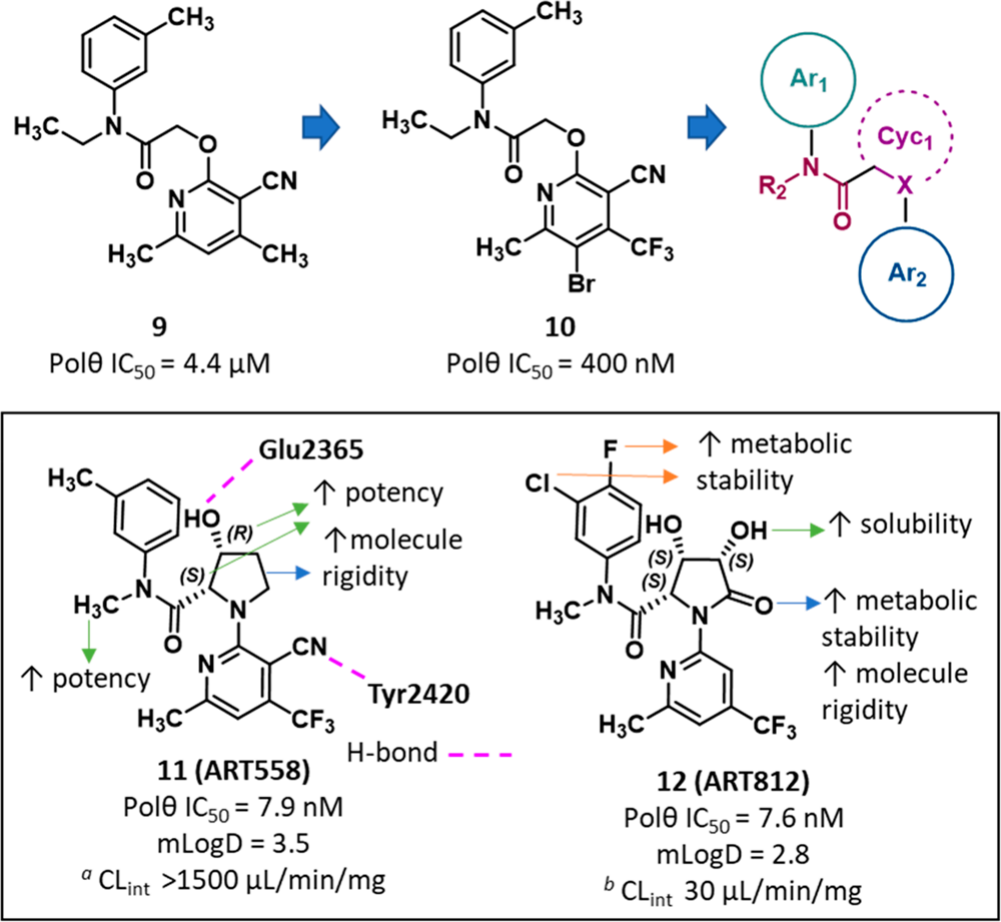
Structure and activity of POLθ-pol inhibitors **ART558**, **ART812**, and analogues.^98^ The IC_50_ value represents the compound concentration that reduces by 50% the POLθ-pol activity as measured by PEA. The CL_int_ value represents the clearance in (*a*) mouse and (*b*) human microsomes.

Among the compounds reported in PA WO2021/028643A1, one compound (compound **11**, **ART558**, Figure 5) was investigated in depth in a recent publication.^95^ Moreover, during the final drafting of this review, Artios reported a follow-up publication describing the medicinal chemistry program that led to the discovery of **ART558** and its analogue **ART812** (Figure 5, and SI, Figure S9).^98^

The authors performed an initial high-throughput screening (HTS), in which ∼165 000 compounds were evaluated by a PicoGreen-based PEA to measure the polymerase activity of full-length (residues 2–2590) POLθ protein expressed in *Escherichia coli*. The screening campaign led to the identification of inhibitors with IC_50_ values in the low micromolar range, among which compound **9** (Figure 5, and SI, Figure S9) was confirmed as a specific hit showing a POLθ IC_50_ of 4.4 μM. Compound **9** did not inhibit PolA and PolH polymerases, was inactive in a POLθ interference assay, and did not bind DNA. Moreover, this small molecule was active in a cellular MMEJ DNA repair nanoluciferase reporter assay at low micromolar concentrations without showing toxicity. Starting from compound **9**, the optimization initially focused on studying different moieties on the pyridine ring, in which lipophilic substituents at the 3- and 4- positions of the pyridine led to increased potency.

Among the synthesized compounds, analogue **10** (Figure 5, and SI, Figure S9, POLθ IC_50_ = 400 nM) was used for cocrystallization studies. A crystal form of POLθ-pol domain (POLθ-PD^Δinsert2^) mutant in complex with DNA and ddGTP was obtained (PDB 7ZUS). Compound **10** was soaked into ternary complex crystals, and the structure was solved at 3 Å resolution (PDB 7ZX0). The results highlighted that this small molecule was able to bind an allosteric site located within the finger subdomain adjacent to the nucleotide binding site. The pocket, which can exist only when the fingers adopted the closed conformation, was created by the movement of the Tyr2412 and Phe2416 side chains induced by the inhibitor binding. The site was highly hydrophobic in nature and composed by 18 predominantly hydrophobic residues. Indeed, compound **10** engaged in extensive hydrophobic interactions within the pocket and, in addition, its nitrile group formed a H-bond with Tyr2420. These data suggested that ligand binding to the allosteric site locks POLθ-pol domain in a closed conformation that prevents incorporation of nucleotides into the DNA primer chain.

Based on the availability of the experimental binding mode of compound **10**, the authors undertook structure-based drug design (SBDD) efforts aimed to further optimize this molecule. The crystallographic ligand conformation underlined the importance of the linker rigidity to allow the correct orientation of the two aromatic rings (Ar_1_ and Ar_2_). Specifically, the presence of the *N*-substitution in the anilide group stabilized a *cis* conformation that was essential for the binding mode of compound **10**. By contrast, the O–CH_2_–C(=O) angle could span a range of values because it is not constrained. Consequently, the ether linker was modified exploiting different amino acids aiming to reduce the flexibility of this portion of the molecule. The best result was obtained by introducing a proline linker that was further decorated with a hydroxyl group in order to decrease the lipophilicity (cLogD ∼ 4). Compound **11** (**ART558**, Figure 5, and SI, Figure S9) showed the best potency with a POLθ IC_50_ of 7.9 nM, a decreased lipophilicity (mLogD = 3.5), and a good thermodynamic solubility of 381 μM. The crystallographic structure of POLθ-PD^Δinsert2^ in complex with **ART558** (PDB 7ZX1) showed that compounds **10** and **ART558** shared a conserved binding pose, but the proline hydroxyl group of **ART558** made an additional H-bond with Glu2365 side chain, which, in concert with the rigidity introduced by the proline linker, may be responsible for its improved potency.

From additional studies, **ART558** showed the best biochemical, cell MMEJ, and phenotypic potency, but biochemical potency improvement did not translate in similar improvement in cells. Thus, further structural optimization of **ART558** was aimed at improving its PK properties, such as trying to increase its very low metabolic stability [intrinsic clearance (Cl_int_) > 1500 μL/min/mg] in mouse liver microsomes. Optimization led to compound **12** (**ART812**, Figure 5, and SI, Figure S9) showing excellent potency (POLθ IC_50_ of 7.6 nM) and reduced lipophilicity (mLogD of 2.8). With respect to **ART558**, **ART812** also displayed an improved metabolic stability (Cl_int_ = 30 μL/min/mg in human hepatocytes) and solubility (880 μM). This new derivative also exhibited good oral bioavailability in both mouse and rat, and moderate-to-high exposure after a single oral dose. Thus, *in vivo* PK/pharmacodynamic studies were performed for **ART812** in order to determine target engagement by evaluating the induction of micronuclei in reticulocytes, which is a marker of DNA damage. After 4-day treatment at the maximum tolerated dose of 150 mg/kg BID, **ART812** showed selective 2-fold increase of micronuclei in reticulocytes with respect to control, as measured by flow cytometry, analogously to POLθ loss in the knockout mouse.

As reported above, before the publication of the medicinal chemistry work that led to the discovery of **ART558** and **ART812**, Artios reported a thorough study in which both compounds were used to demonstrate the potential clinical utility of POLθ inhibitors.^95^ Notably, initial studies aimed at determining the mechanism of action, selectivity, and target engagement of **ART558** were performed. In particular, mechanistic studies were performed for **ART558**, showing a noncompetitive inhibition with respect to dNTPs and uncompetitive inhibition with respect to DNA, suggesting an allosteric binding site within the POLθ-pol catalytic domain, as later confirmed by crystallographic studies.^98^ Moreover, **ART558** induced POLθ thermal stabilization, but only in the presence of DNA, as shown by using differential scanning fluorimetry (DSF).

Interestingly, **ART558** did not inhibit other human DNA polymerases (Polα, Polγ, Polη, and Polν) and showed no significant inhibition of 78 oncology-relevant kinases as well as PARP1 and PARP2 at 10 μM. Furthermore, **ART558** inhibited POLθ-mediated DNA DSB repair TMEJ in a dose-dependent manner (EC_50_ = 150 nM, PCR and an adapted luminescence-based DNA reporter assays), but it was unable to inhibit canonical NHEJ, thus demonstrating its excellent selectivity. **ART558** specificity was also demonstrated by its ability to only enhanced radiosensitivity in *POLQ* wild-type cells while having no effect in *POLQ* null cells, suggesting epistasis with *POLQ* deletion and an on-target effect.

Analogously to genetic inactivation of *POLQ* that confers SL in *BRCA2* gene defective cells, **ART558** sensitivity was observed in genetically engineered *BRCA2*^*–/–*^ cells (PARPi sensitive) compared to isogenic *BRCA2*^*wt*^ cells (PARPi resistant). Moreover, **ART558** plus the PARPi **olaparib** showed a far greater effect on cell survival, culture confluency, and apoptosis in *BRCA2*^*–/–*^ cells than in *BRCA2*^*wt*^ cells. **ART558**-induced SL and a combination effect with **olaparib** were also observed in isogenic models of *BRCA1*-deficient cells, and **ART558** sensitivity was observed in tumor cell lines with pathogenic *BRCA1* mutations, suggesting that these effects are not specific to *BRCA2* mutant cells, but also extend to *BRCA1* mutant tumor cells. Importantly, cell growth inhibition of *BRCA1* or *BRCA2* mutant cells was achieved at **ART558** concentrations that had minimal effects in nontumor epithelial cells or *BRCA2*^*wt*^ tumor cells.

**ART558** sensitivity was observed in an *ex vivo* cultured tumor organoid derived from a *BRCA1* mutant breast cancer, but not in a *BRCA1*^*wt*^ breast cancer organoid cultured under similar conditions.

Profound **ART558** sensitivity was caused in *BRCA1*^*–/–*^ cells by siRNA targeting *MAD2L2* (aka *REV7*) and *SHLD2* (*FAM35A*), which encode components of the MAD2L2/SHLD1/SHLD2/SHLD3 “Shieldin” complex. This complex prevents DNA resection at DSBs and, importantly, loss of Shieldin components causes PARPi resistance in *BRCA1* mutant cells. However, **ART558** sensitivity was not caused by siRNA targeting MAD2L2 and SHLD2 in *BRCA1*^*wt*^ cell, suggesting that combined defects in *BRCA1* and the Shieldin complex could be associated with **ART558** sensitivity.

Due to poor *in vitro* metabolic stability in rat microsomes shown by **ART558**, **ART812** was used to determine the ability of these compounds to target established *BRCA1/SHLD2* defective tumors *in vivo* (rats bearing established MDA-MB-436 *BRCA1*/*SHLD2* defective tumors) because of its better PK profile. Dosing rats with **ART812** resulted in significant tumor inhibition and was well tolerated.

The NHEJ factor 53BP1 (encoded by *TP53BP1*) recruits Shieldin components to DSBs and loss of *53BP1*, *SHLD1* and *SHLD3* causes PARPi resistance in *BRCA1* mutant cells. PARPi resistant SUM149 clones generated with either *SHLD1*, *SHLD3*, or *53BP1* mutations exhibited **ART558** sensitivity while being resistant to **olaparib**. Moreover, while *BRCA1* mutation produced a moderate increase in **ART558** sensitivity, and *TP53BP1* mutation alone did not cause **ART558** sensitivity, the combination of both mutations was associated with profound **ART558** sensitivity. Additionally, cells of a tumor patient-derived *ex vivo* culture, with low BRCA1 and 53BP1 expression, were resistant to **olaparib** or **carboplatin** but sensitive to **ART558**. In assessing the mechanistic basis of these findings, the authors proposed that the absence of 53BP1/Shieldin function leads to an increase in resection and ssDNA when cells are exposed to a POLθ inhibitor, where the resected DNA ends are presumably repaired by a BRCA1-mediated process. On the other hand, when both BRCA1 and 53BP1/Shieldin function are impaired, POLθ becomes essential for repairing resected ssDNA caused by the exposure of DSB ends to nucleases due to Shieldin loss; in these cells, POLθ inhibition leads ultimately to SL. Finally, **ART558** increased biomarkers of ssDNA and SL in 53BP1-defective cells, while the inhibition of DNA nucleases that promote end-resection reversed these effects, suggesting their involvement in the SL mechanism-of-action.

The results of this study were reported in the PA WO2021/028644A1^102^ filed by Artios in 2019. In particular, the PA reported that the inhibition of POLθ is SL to cancer cells that are resistant to PARP inhibition through loss of Shieldin components, a finding with significant implications for the treatment of cancer patients bearing tumors that are resistant to PARPi -based therapies.

In August 2022, Artios announced to have initiated a Phase 2 study with **ART4215** (undisclosed structure) in combination with the oral PARPi **talazoparib** (TALZENNA).^118,119^**ART4215** is the first oral small molecule POLθ inhibitor to enter clinic trials.

Preclinical studies have demonstrated that **ART4215** may have broad potential clinical utility. Currently, this drug candidate is being evaluated in a first-in-human, global, open-label Phase 1/2 study to assess its safety, tolerability, PKs, and clinical activity as a monotherapy or in combination with **talazoparib** in patients with advanced or metastatic solid tumors. Initial safety and tolerability data from the first Phase 1 dose cohorts demonstrated that **ART4215** was well tolerated. Results from Phase 1 are expected in the first half of 2023. Based on these data, a Phase 2 study (NCT04991480) has been initiated. In particular, a recommended Phase 2 dose has been established for **ART4215** in combination with **talazoparib**. Moreover, a randomized expansion cohort has been initiated to evaluate the combination in patients with *BRCA*-deficient breast cancer. The study is enrolling up to 206 patients and is conducted at multiple oncology centers across Europe and the USA. Phase 2 data are expected in August 2025.

#### 2.1.2 POLθ-pol Inhibitors RP-6685

**Figure 6:**
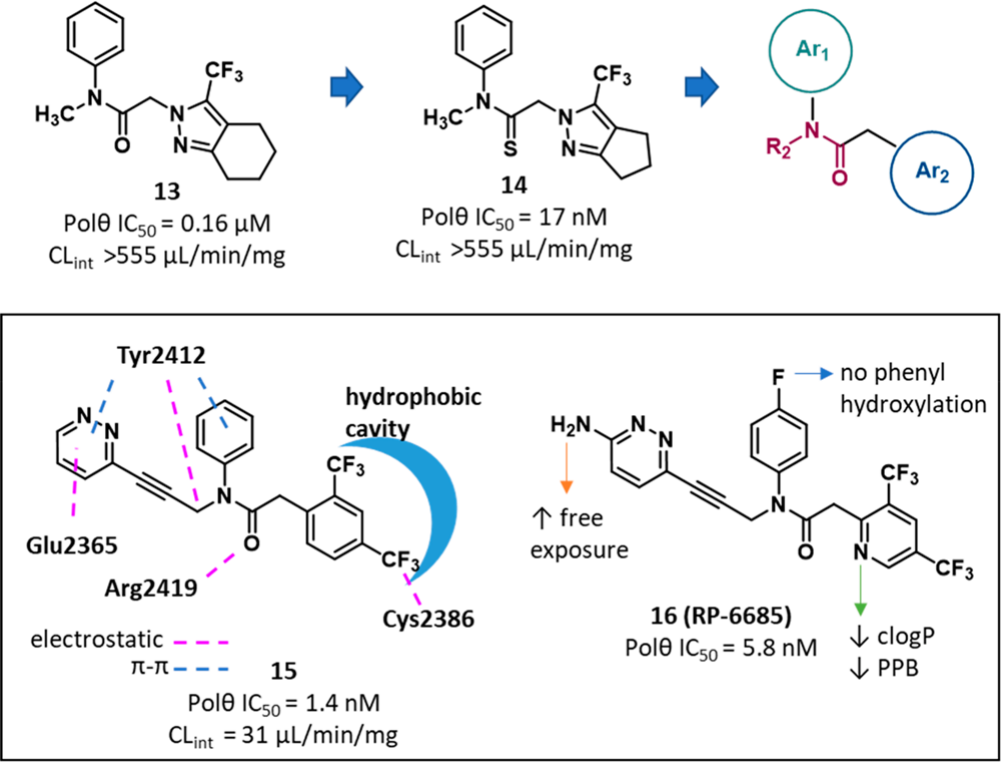
Structure and activity of the POLθ-pol inhibitor **RP-6685** and analogues.^97^ The IC_50_ value represents the compound concentration that reduces by 50% the POLθ-pol activity as measured by PEA. The CL_int_ value represents the clearance in mouse microsomes.

Very recently, Repare Therapeutics reported a new series of POLθ-pol inhibitors.^97^ Also in this case, the pharmaceutical company used HTS to test 350 000 compounds in a DNA PEA PicoGreen-based assay using POLθ-pol domain (residues 1819–2590) expressed in *E. coli*. The screening led to identify a mixture of compound **13** (Figures 6, and SI, Figure S10) and its N1-regioisomer, showing, once isolated, IC_50_ values of 0.16 and 11 μM, respectively. Based on its potency, compound **13** was selected as a hit for follow-up, which was mainly targeted to optimizing ADME properties. The first optimization efforts were focused on the 3-(trifluoromethyl)-4,5,6,7-tetrahydro-2*H*-indazol-2-yl ring, and in one compound, the acetamide oxygen was replaced by a sulfur atom, to give derivative **14** (Figure 6, and SI, Figure S10). Compound **14** exhibited an improved anti-POLθ-pol activity (IC_50_ = 17 nM) but the same low microsomal stability of compound **13**. Biophysical studies showed that compound **14** alone did not induce any thermal stabilization of WT POLθ, as shown by DSF, while a compound-based thermal shift was observed in the presence of a dsDNA substrate providing a 5′ overhang, which mimics the primer:template pair, indicating an uncompetitive mechanism of action. Moreover, kinetic studies indicated a noncompetitive inhibition with respect to dNTPs, thus suggesting for compound **14** a binding site within the POLθ-pol domain different from the nucleotide-binding site. These results were further confirmed by X-ray crystallography studies, in which compound **14** was cocrystallized with an engineered construct of POLθ-pol (truncations in the protein expression construct were engineered by deleting five flexible loops that were not resolved in previously published structures) in complex with dsDNA and ddGTP (PDB 8E23). An allosteric site located in the finger subdomain opposite to the binding position of the incoming ddGTP emerged as the binding site of compound **14**. Of note, compound **14** and **ART** compounds previously described shared the same allosteric binding site.

Based on the binding mode of compound **14**, a SBDD approach was performed, leading to design compound **15** (Figure 6, and SI, Figure S10), which was cocrystallized with the engineered construct of POLθ-pol (PDB 8E24). The solved ligand–protein complex showed that (i) the Tyr2412 side chain interacted with the Ar_1_ benzene ring and the R_2_ pyridazine ring (which extended out from one end of the solvent exit channel) through π–π stacking interactions, (ii) the backbone carbonyl of Tyr2412 bound the partially positive R_2_ methylene group through an electrostatic interaction, (iii) electrostatic interactions were also present between the Glu2365 carboxylate and a pyridazine carbon atom, and the Arg2419 guanidinium group and the amide carbonyl, and (iv) the Ar_2_ 2,4-bis(trifluoromethyl)phenyl moiety filled a hydrophobic cavity, with the *para*-CF_3_ substituent establishing an electrostatic interaction with Cys2386 sulfur atom. Final optimization of compound **14** was aimed to improve its PK properties, focusing on the free exposure [total exposure in mouse plasma corrected for plasma protein binding (PPB)]. Compound **16** (**RP-6685**, Figure 6, and SI, Figure S10) showed the best profile, with an IC_50_ of 5.8 nM.

From a structural point of view, **RP-6685** showed some similarities with compounds previously described (Figures 3 and 5). In particular, the two aromatic systems (Ar_1_ and Ar_2_) connected by a central linker were present, even though the four-atoms linker (N–C(=O)–C–X) characterizing all the previous POLθ-pol inhibitors was shortened by deleting the X atom in the **RP-6685** series. On the other hand, **RP-6685** was characterized by a more extended and bulkier R_2_ substituent, with respect to previous compounds; the R_2_ substituent was explored in depth in this work proving to have a key role in imparting potency to the compound.

**RP-6685** was further characterized showing high selectivity for POLθ, being inactive against human DNA polymerases α, ε, γ, λ, and μ. Moreover, this small molecule potently inhibited the polymerase activity of full-length POLθ produced in HEK293 cells (IC_50_ = 550 pM) but not the ATPase activity. On-target activity of **RP-6685** was also shown in a DSB assay in which CRISPR/Cas9-mediated break was induced at the AAVS1 locus in HCT116 cells (DSB can proceed by NHEJ and MMEJ): **RP-6685** inhibited the MMEJ with an IC_50_ of 0.5 μM. Moreover, **RP-6685** showed a dose-dependent decrease (IC_50_ = 0.94 μM) of MMEJ in a traffic-light reporter assay in HEK293 *LIG4*^*–/–*^ cells, which are unable to repair DSBs by NHEJ (DSB can proceed by MMEJ and HR).

When evaluated in a cell proliferation assay employing isogenic HCT116 *BRCA2*^*–/–*^ and *BRCA2*^*+/+*^ cell lines, **RP-6685** showed a ca. 50-fold decrease in potency in killing *BRCA2*^*–/–*^ cells (IC_50_ values of 0.32 and >15 μM, respectively), with respect to the potency observed in the biochemical assay. Accordingly, **RP-6685** treatment (oral administration at 80 mg/kg BID for 21 days) did not inhibit tumor growth in a *BRCA2*^*+/+*^ HCT116 mouse xenograft but tumor regression was observed in the *BRCA2*^*–/–*^ HCT116 xenograft during the first 8 days of treatment, although it was not sustained throughout the treatment period. PK studies showed that **RP-6685** exposure declined by 55–67% over a week of dosing, possibly due to induction of metabolizing enzymes. Finally, pharmacodynamics studies performed on a *BRCA2*^*–/–*^ HCT116 xenograft treated with **RP-6685** showed a modest trend toward increased micronuclei and γH2AX, which are hallmarks of DNA damage.

#### 2.1.3 Common Features of POLθ-pol Allosteric Inhibitors

The amount of chemical and biological data collected for the different chemical series of POLθ-pol inhibitors allowed us to derive some general considerations, such as the definition of the basic chemical structure for compounds reported in Figures 3, 5, and 6. We have also attempted to use the available information to delineate a structure–activity relationship (SAR) for these inhibitors, with the goal of determining the key chemical features responsible for the biological activity *in vitro*.

Achieving this objective presented a number of issues, in particular: (i) many PAs reported compounds that were annotated with activity ranges rather than the individual IC_50_ values, (ii) derivatives described in the PAs show comparable activity, with most of them having IC_50_ values ranging from 2 to 200 nM and only a few molecules displaying IC_50_ = 1–10 μM, and (iii) some PAs reported a very large number of analogues. These limitations aside, we have been able to derive some relevant observations. First, regardless of the chemical series, the enzymatic inhibition assay used to evaluate the potency of each molecule on the isolated target was consistent across PAs. Second, the recently published manuscripts describing the identification, optimization, and cocrystallization of POLθ-pol **ART** and **RP** inhibitors, which bound to the same allosteric pocket, provided a valuable contribution to better understand the mechanism of action of these inhibitors and the role of some chemical features in modulating the binding to POLθ or the PK properties.

**Figure 7:**
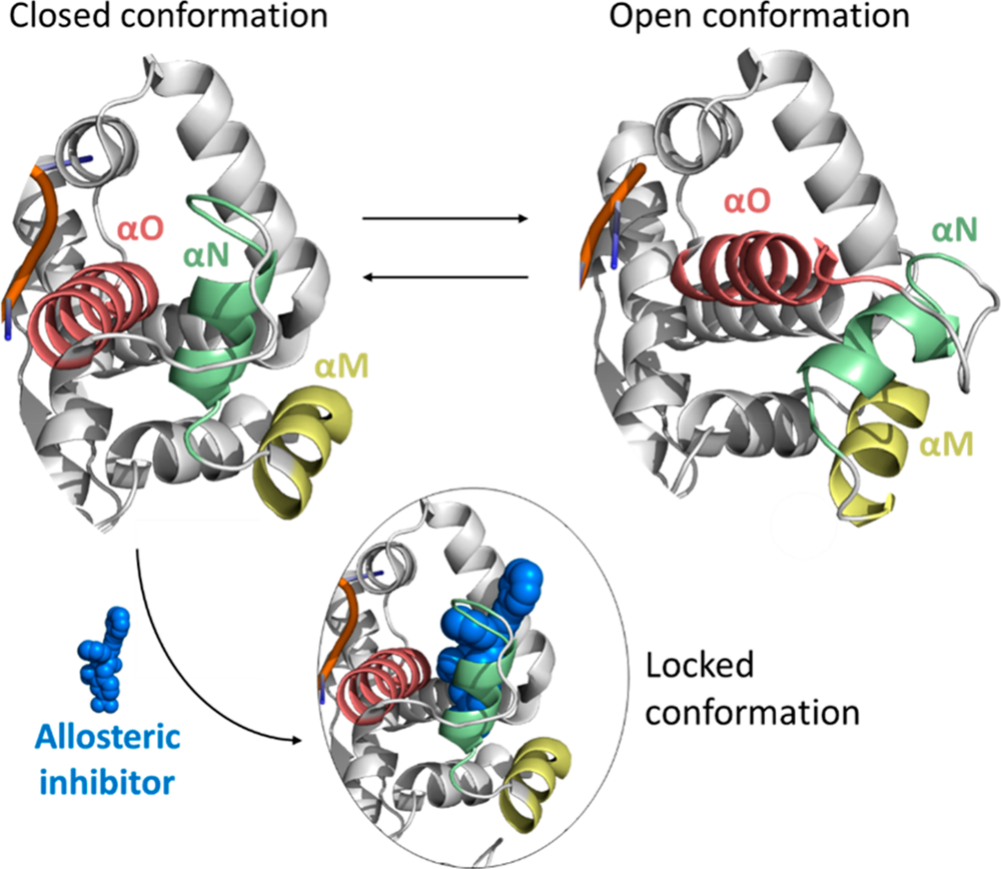
Movement of the three helixes of the finger domain (helix αM, helix αN, and helix αO) involved in the switch from the closed to the open conformation and mechanism of action of allosteric inhibitors.

Regarding the mechanism of action, the available crystallographic structures suggested that the allosteric inhibitors **ART558**, **ART812**, and **RP-6685** were able to interfere with the catalytic cycle of DNA synthesis, during which the finger domain adopted two main conformations (Figure 7). Specifically, a close conformation of the finger domain stabilized the binding of both the nucleic acid and the incoming nucleotide to the enzyme. Following the incorporation of the ddNTP to the nascent chain, an open conformation was adopted by the finger domains to allow the translocation of the DNA and the release of the pyrophosphate from the active site. The inhibitors acting on this pocket locked the finger domain in the closed state (Figure 7), hampering the switch to the open state and causing the block of the cycle. Notably, the fingers domain acquired a closed conformation only when the protein is bound to the nucleic acid, thus explaining the observed nucleic acid dependent activity for this POLθ allosteric inhibitors.

**Figure 8:**
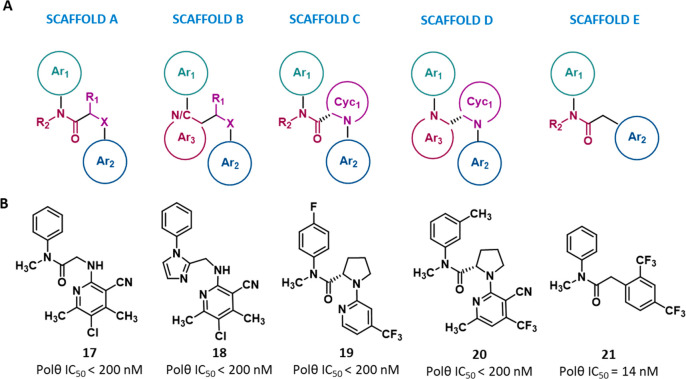
(A) Scaffolds summarizing the chemical series belonging to the same class of allosteric POLθ-pol inhibitors; for each scaffold, the general chemical features are reported. (B) Representative compound for each scaffold, with the corresponding IC_50_; the IC_50_ represents the compound concentration necessary to reduce by 50% the POLθ-pol activity as measured by PEA.

Finally, the detailed analysis performed on all the chemical series of POLθ-pol inhibitors reported so far led us to summarize their structures into five different scaffolds (Figure 8A), which clearly shared some common chemical features, i.e., Ar_1_, Ar_2_, and the linker.

Thanks to the availability of homogeneous and therefore comparable biological data, we have focused our attention on the most potent inhibitors of all chemical families (IC_50_ ≤ 200 nM; activity class “++++” in PAs) to highlight important characteristics of the three essential chemical groups, thus providing some SAR insights.

Specifically, the **Ar**_**1**_**group** was always represented by a 6-membered (hetero)aromatic ring and, in particular, by a phenyl, a pyridine, or a pyrimidine. The decoration of this moiety was not essential for activity. Indeed, even though potent inhibitors were in most cases decorated with a large variety of substituents of different size (e.g., H, F, Cl, Me, OCF_3_, CN, vinyl, and cyclopropyl) and mainly hydrophobic in nature, high activity compounds having an unsubstituted Ar_1_ moiety were also present (Figure 8B). By contrast, the modification of this moiety was generally aimed at improving the compound PK properties. For example, many of the reported compounds were characterized by a fluorine atom at the *p*-position of the Ar_1_ benzene ring (often associated with a chlorine atom at the *m*-position) that prevented phenyl hydroxylation, thus increasing the microsomal stability.

The Ar_1_ moiety was connected to Ar_2_ by a **linker**, which appeared to be the most variable part of the molecules in terms of chemical modifications introduced. We considered the linker as composed by a planar portion and a nonplanar one. In scaffolds A, C, and E, the planar portion is made of an *N*-substituted amide motif, while in scaffolds B and D, it is embedded in a 5- or 6-membered aromatic ring (Ar_3_). It is noteworthy that, in the former series, all compounds showed a small *N*-alkyl substituents (R_2_). As demonstrated by crystallographic data, this peculiar feature promoted a *cis* amide conformation necessary for the correct orientation of Ar_1_ and Ar_2_ in the allosteric binding site. Indeed, in scaffolds B and D, the amide linker was replaced by an aromatic ring system that located the two principal substituents in a *cis* conformation. Additionally, in scaffold E, the extensive exploration of the *N*-substitution (R_2_) permitted both to improve compounds activity up to 200-fold, thanks to additional interactions with the binding site, and to modulate the PK properties.

The nonplanar portion of the linker was characterized by a single sp^3^ carbon in scaffold E, while in scaffolds A–D, it always corresponded to a sp^3^ carbon (C*) bounded to a heteroatom (X represented by a N, O, or S). The C*–X system could be either a linear system, as in scaffolds A and B or embedded in a ring system (Cyc_1_) as in scaffolds C and D.

However, unlike Ar_3_, Cyc_1_ was always a saturated heterocyclic ring, with a chiral center corresponding to the C* position. Interestingly, the (*S*)-stereochemistry at the C* center in the heterocyclic ring is associated with potent inhibition of the enzyme, as opposed to what was observed in compounds having the (*R*)-stereochemistry. Finally, the **Ar**_**2**_**group** was always represented by an (hetero)aromatic ring that could also be fused with a second 5- or 6-membered aliphatic or aromatic ring. The number and type of substitutions on Ar_2_ seemed to be more conserved than the reported substitutions on Ar_1_, with an overrepresentation of trifluoromethyl, methyl and nitrile groups decorating a pyridine ring. In this context, the pyridine ring was preferred over the benzene to increase the hydrophilicity,^97^ the *p*-trifluoromethyl moiety could interact with Cys2386 through electrostatic contacts (as shown by compound **15**-POLθ-pol crystal structure^97^), while the nitrile moiety appeared to contribute to the conformational restriction of Ar_2_ orientation (as shown for **ART** compounds^98^). Analogously to Ar_1_, potent compounds were characterized by large and hydrophobic groups at Ar_2_.

To gain further insight into the minimal pharmacophore required for POLθ inhibitory activity and to aid in the rational design of novel POLθ allosteric ligands, we have developed a 3D ligand-based pharmacophore model using one representative compound of each scaffold as training set (Figure 8B).

**Figure 9:**
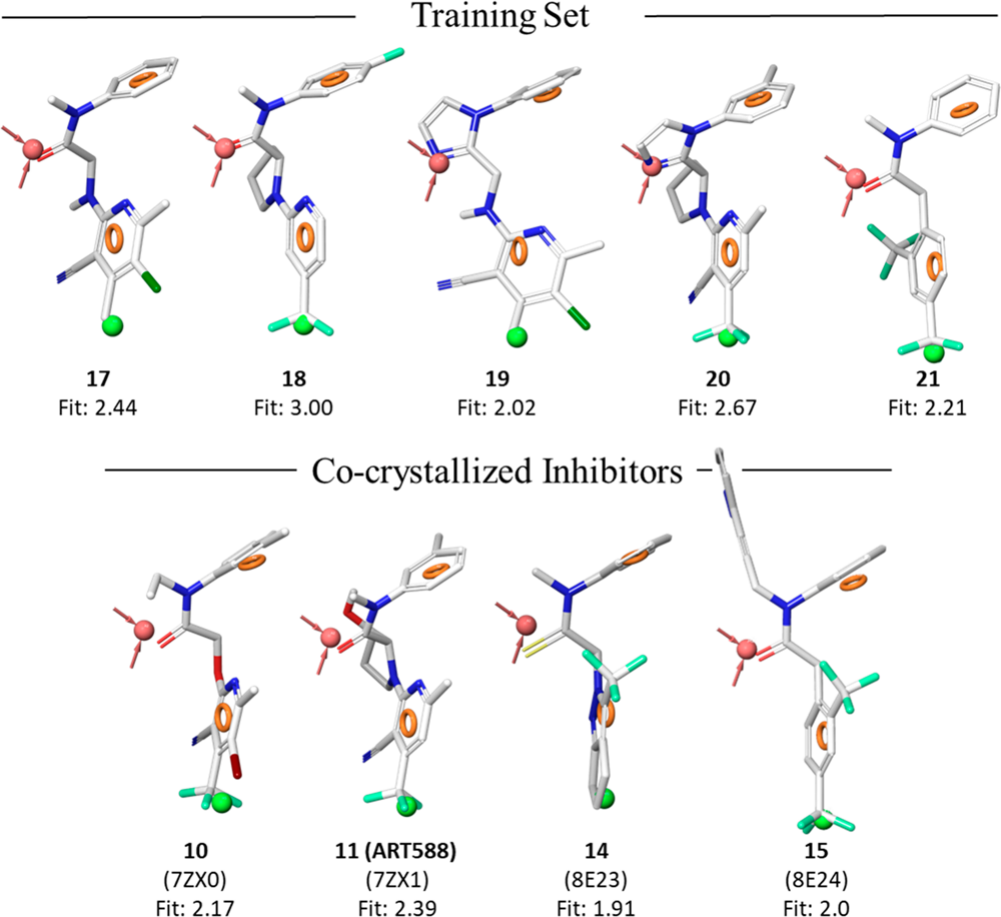
Fitting of the training set compounds and cocrystallized inhibitors on the 3D-pharmacophore model developed using Phase (Schrödinger suite). Red sphere and vectors, H-bond acceptor; blue sphere, positive charge; orange ring, aromatic ring; green sphere, hydrophobic moiety. The Fitness value (Fit) measures how well the ligand conformer matches the pharmacophore hypothesis. A perfect alignment corresponds to a fitness score of 3.

The final model (Figure 9, see Supporting Information and Table S1 for additional details) was characterized by four features represented by two aromatic portions, corresponding to Ar_1_ and Ar_2_, one hydrophobic region relating to a substituent on the Ar_2_ ring and one acceptor group placed in the planar portion of the linker. Although the presence of an H-bond acceptor as a pharmacophore feature did not emerge from the crystallographic data of ligand–receptor complexes available to date, this intermolecular interaction cannot be ruled out given the protein plasticity and/or the possibility of water-mediated hydrogen bonding. Interestingly, in all compounds collected, the acceptor feature overlapped with an sp^2^-hybridized heteroatom, in which case this pharmacophore element also became an indicator of the presence of an atom contributing to linker planarity.

It is noteworthy that all the cocrystallized ligands in their experimental binding modes were able to properly fit the 3D model (Fitness > 1.9, Figure 9), thus validating this *in silico* tool and supporting its potential use in future virtual screening campaigns for the identification of new POLθ-pol allosteric inhibitors.

### 2.2 POLθ Helicase Domain Inhibitors

**Figure 10:**
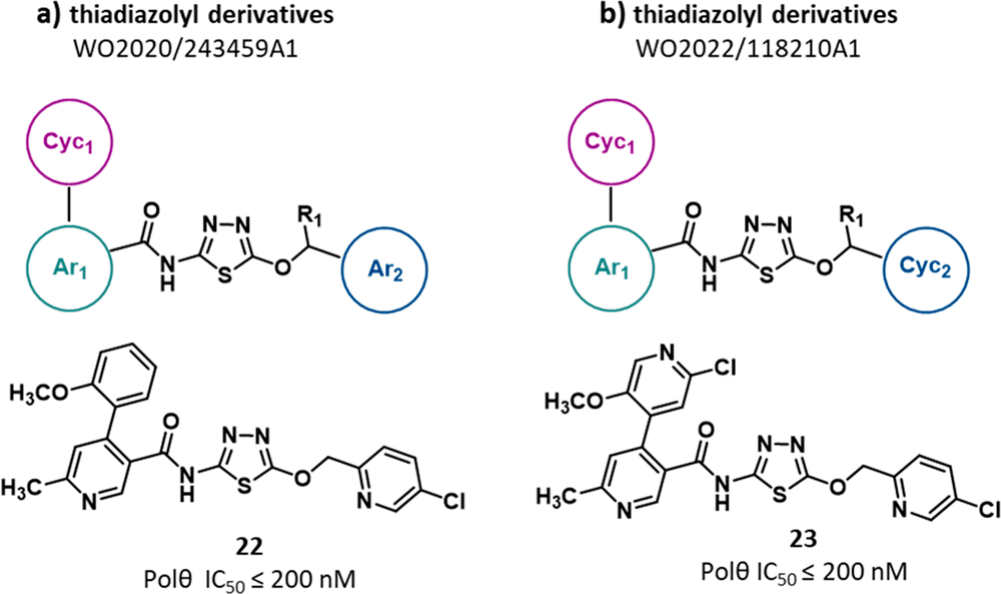
Structure of thiadiazolyl derivatives (a,b) as POLθ-hel inhibitors reported in PAs filed by Ideaya.^113,114^ The IC_50_ value represents the compound concentration that reduces by 50% the POLθ-hel activity as measured in the NADH oxidation-coupled enzymatic assay.

From 2020 to 2021, Ideaya filed two PAs reporting POLθ-hel domain inhibitors characterized by a thiadiazolyl scaffold. PA WO2020/243459Al,^113^ filed in 2020, reported 232 **thiadiazolyl derivatives** (exemplified by compound **22**, Figure 10a, and SI, Figure S11) characterized by a central *N*-(5-methoxy-1,3,4-thiadiazol-2-yl)acetamide portion linking two (hetero)aromatic moieties, Ar_1_ and Ar_2_, of which Ar_1_ was directly bound to a heterocycle (Cyc_1_). For 224 compounds out of the 232 reported in the PA, their ability to inhibit the ATPase activity of POLθ was determined in a NADH oxidation-coupled enzymatic assay by measuring the rate of the ATP turn over. In this assay, the POLθ-hel domain (residues 1–899) expressed in baculovirus was used. As far as the inhibitory activity is concerned, no individual IC_50_s were reported and compounds were assigned to four groups depending on their potency (IC_50_ = 10 μM to 1 μM, = 1 μM to 500 nM, = 500 nM to 200 nM, and <200 nM). Specifically, 136 compounds showed an IC_50_ < 200 nM, while 36 compounds displayed an IC_50_ in the 1–10 μM range.

In 2021, Ideaya filed the most recent PA on POLθ inhibitors reported to date (WO2022/118210Al^114^). The patent discloses 273 additional **thiadiazolyl derivatives** (exemplified by compound **23**, Figure 10b, and SI, Figure S12), strictly related to those previously reported. In particular, also in these compounds the central *N*-(5-methoxy-1,3,4-thiadiazol-2-yl)acetamide portion is linked to an aryl moiety (Ar_1_) that, in turn, is connected to an aliphatic or aromatic cycle (Cyc_1_). The central portion is also linked to a second aliphatic or aromatic cycle (Cyc_2_), differently from compounds reported in Figure 10a, which are characterized by an aromatic Ar_2_ substituent in the same position. Analogously to compounds described before, the ability of compounds to inhibit the POLθ ATPase activity was evaluated by a NADH oxidation-coupled enzymatic assay and compounds were gathered in different groups based on their inhibitory potency (from 10 μM to <200 nM). Out of the 273 reported compounds, 243 showed potent activity with an IC_50_ value <200 nM, while only 10 displayed an IC_50_ in the 1–10 μM range. For both PAs, no activity in cellular assays has been described for any compound and no follow-up papers have been published at this time.

In 2022, Ideaya announced selection of a potential first-in-class POLθ-hel development candidate (undisclosed structure).^120^ Ideaya and GlaxoSmithKline (GSK) are collaborating on the ongoing IND-enabling studies to support the evaluation of their small molecule in combination with **niraparib**, a GSK’s PARPi, for patients having tumors with BRCA or other HR mutations or deficiency. The development candidate in combination with **niraparib** was reported to have robust *in vivo* efficacy, with significant tumor regressions and durable responses in multiple cancer models. Subject to satisfactory completion of ongoing preclinical and IND-enabling studies, Ideaya and GSK are targeting an IND submission of the clinical candidate, allowing first-in-human studies, in the first half of 2023.

Dana-Farber filed in 2016 a first PA (WO2017/070198A1^105^) reporting the discovery of a SL relationship between HR-deficiency and POLθ blockade, a method for treating HR-deficient cancers (also those resistant to PARPi), and a high-throughput screening approach for identifying DNA polymerase ATPase activity inhibitors. Then, from 2018 to 2020, the Institute filed three additional PAs, in which small molecules POLθ ATPase activity inhibitors were disclosed.

PA WO2019/079297A1^106^ (filed in 2018), provided a method for using **2-oxo-2***H***-chromene**, **naphthalene**, and **quinoline derivatives** in treating HR-deficient cancer. In particular, the disclosure provided a method for: (i) determining that the HR-deficient cancer contains a mutation or an alteration in a gene regulating homologous recombination, (ii) treating HR-deficient cancer by administering a therapeutically effective amount of a claimed compound alone or in combination with an anticancer agent, i.e., platinum-based compounds or PARPi, and (iii) inhibiting POLθ in a cancer cell.

**Figure 11:**
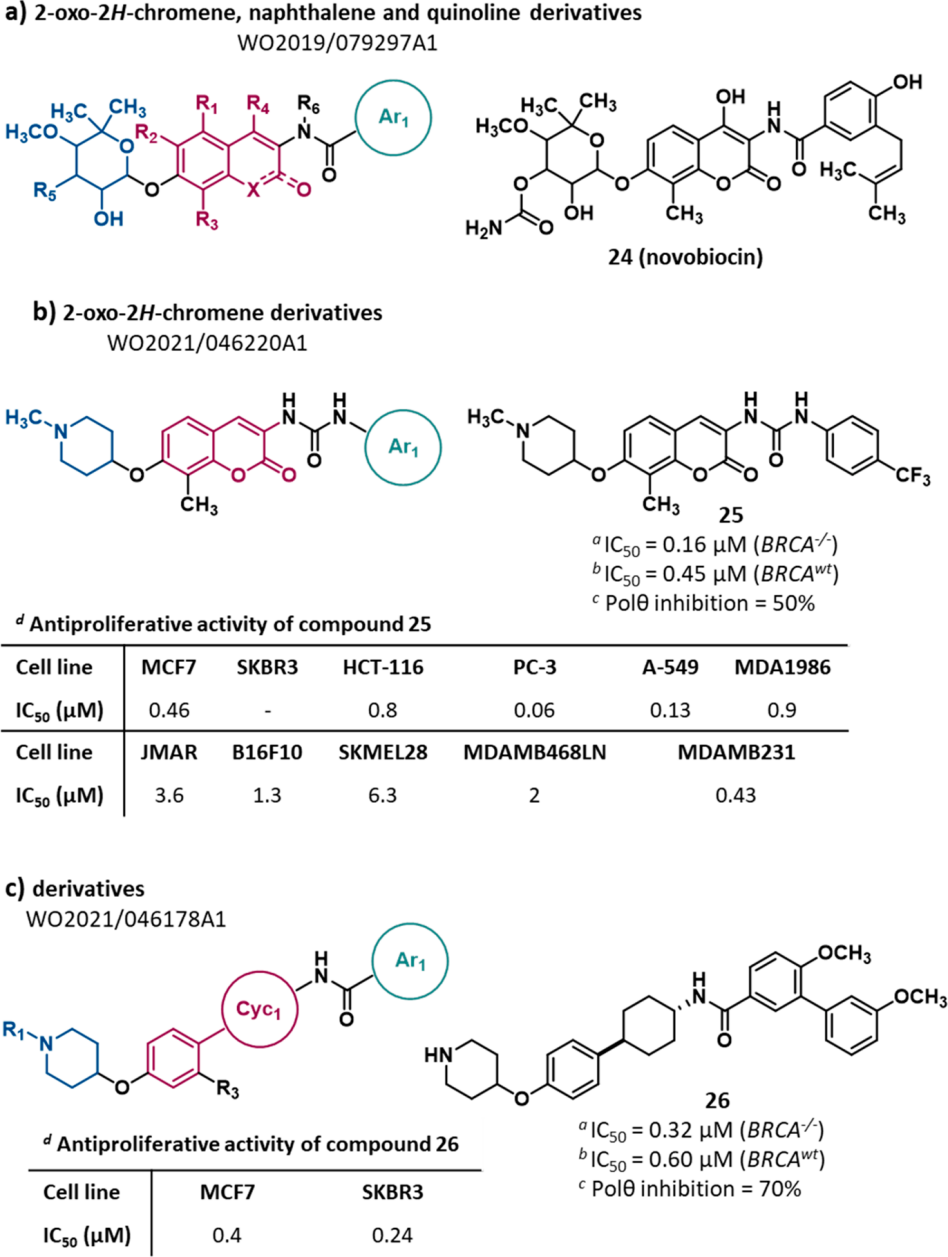
Structure of 2-oxo-2*H*-chromene, naphthalene, and quinoline derivatives^106^ (a), 2-oxo-2*H*-chromene derivatives^107^ (b), and derivatives^108^ (c) as POLθ-hel inhibitors reported in PAs filed by Dana-Farber. The IC_50_ value represents the compound concentration that reduces by 50% cell viability (CTG cell viability assay) in (*a*) *BRCA1*^*–/–*^ and (*b*) *BRCA1*^*wt*^ RPE1 cells. (*c*) Percentage of POLθ ATPase activity inhibition determined by ADP-Glo ATPase assay. (*d*) The IC_50_ value represents the compound concentration that reduces by 50% cell proliferation.

Focusing on the 24 derivatives reported, 20 were characterized by a 2-oxo-2*H*-chromene nucleus exemplified by compound **24** (**novobiocin**, **NVB**), while four by a naphthalene or a quinoline core (Figure 11a, and SI, Figure S13). Among the reported compounds, **NVB** was the only derivative evaluated in cells. Since the results are also described in a recent publication^96^ reporting an in-depth characterization of the compound, they are described below (see section 2.2.1.).

In 2020, Dana-Farber filed two PAs reporting **NVB** analogues. In particular, PA WO2021/046220Al^107^ reported 27 **2-oxo-2***H***-chromene derivatives** (exemplified by compound **25**, Figure 11b, and SI, Figure S14). In these compounds, a 1-methylpiperidin-4-yloxy moiety and a methyl group decorated the C-7 and C-8 positions of the scaffold, respectively. Moreover, an urea moiety linked the core to a mono- or disubstituted phenyl ring. The percentage of POLθ ATPase activity inhibition of 14 compounds was measured in an ADP-Glo ATPase assay: an inhibition of enzyme activity in the range 10–50% was observed for all but one derivative, which showed 80% inhibition. The ability to kill *BRCA1*^*–/–*^ versus *BRCA*^*wt*^ RPEl cells was evaluated for 18 compounds by employing the luminescence-based Rapid Cell Titer Glow (CTG) cell viability assay. Compounds showed IC_50_s ranging from 0.16 to 3.73 μM in *BRCA1*^*–/–*^ RPE1 cells (resulting 200 times more potent than **NVB**, having an IC_50_ = 86 μM) and from 0.42 to 5.33 μM in *BRCA*^*wt*^ RPE1 cells: the highest selectivity ratio observed was 2.75. For three compounds (at 100 μM concentration), the ability to kill *BRCA1*^*–/–*^ RPE1 cells was also confirmed in a clonogenic survival assay (CSA). The same compounds also resulted more effective in killing *BRCA1*-mutated cancer cell lines (MDA-MB-436 and UWB1) than their WT counterparts (MDA-MB-436 + BRCAl and UWBl + BRCA1) in the CSA, demonstrating that the compounds induce SL with HR. Since **NVB** was reported to inhibit Hsp90 protein, 22 compounds were evaluated for this activity: 17 compounds acted as selective POLθ inhibitors while 5 derivatives were dual inhibitors of POLθ and Hsp90. Additionally, the antiproliferative activity of 24 compounds was evaluated in a panel of 11 cancer cell lines (as example, activity of compound **25** was reported in Figure 11b). The remaining three derivatives were assayed in *P53*^*–/–*^- and *P53*^*–/–*^*-BRCA1*^*–/-*^ RPE1 cells by CTG cell viability assay, resulting in IC_50_ values ranging from 0.04 to 0.11 μM in *P53*^*–/–*^ RPE1 cells (**NVB** showed an IC_50_ of 358.4 μM) and from 0.008 to 0.05 μM in *P53*^*–/–*^*-BRCA1*^*–/–*^ RPE1 cells (**NVB** showed an IC_50_ of 97.24 μM).

Regarding PA WO2021/046178Al,^108^ 14 derivatives were disclosed (exemplified by compound **26**, Figure 11c, and SI, Figure S15), in which the 2-oxo-2*H*-chromene scaffold was replaced by a phenyl ring linked to an additional phenyl or aliphatic cycle (Cyc_1_). Analogously to **NVB**, an amide moiety (only one compound showed a urea moiety as a linker) linked Cyc_1_ to an (hetero)aromatic ring (Ar_1_). The percentage of POLθ ATPase inhibition determined by ADP-Glo ATPase assay was reported only for two compounds (70% and 30%), while 10 derivatives were assessed for their ability to kill *BRCA1*^*–/–*^ versus *BRCA1*^*wt*^ RPEl cells (luminescence-based CTG cell viability assay). Compounds showed IC_50_ values comparable to those of the analogues previously mentioned, ranging from 0.32 to 5.56 μM in *BRCA1*^*–/–*^ RPE1 cells and from 0.59 to 7.95 μM *BRCA1*^*wt*^ in cells, the highest selectivity ratio being 2.8. Additionally, the antiproliferative activity of the 14 small molecules was evaluated in a panel of 11 cancer cell lines, but results were disclosed only for 10 compounds in the MCF7 and SKBR3 cell lines, with IC_50_s ranging from 0.4 to 5.1 μM and from 0.24 to 5.2 μM, respectively (as example, activity of compound **26** was reported in Figure 11c). For the remaining four derivatives, the antiproliferative activity in *P53*^*–/–*^- and *P53*^*–/–*^*-BRCA1*^*–/–*^ RPE1 cells, measured in the CTG cell viability assay, was reported, with IC_50_ values of 0.04–0.2 μM and 0.05–0.5 μM, respectively.

#### 2.2.1 POLθ-hel Inhibitor NVB

Analogously to POLθ-pol inhibitor **ART558**, POLθ-hel inhibitor **NVB** was used to investigate the relevance of targeting POLθ in cancer, and the results were reported in a recent publication.^96^ NVB is a well-known antibiotic able to bind to the ATP-binding pocket of DNA gyrase and inhibit ATP hydrolysis. Also in this case, the study started with a HTS in which 23 513 small molecules were evaluated as POLθ ATPase inhibitors based on ADP-Glo luminescent assay. Significant reduction of POLθ ATPase activity was shown by 72 compounds, which were further evaluated in presence and absence of ssDNA, in order to exclude compounds that directly interacted with ssDNA. Ten small molecules showed more than 50% inhibition both with and without ssDNA with a difference ≤15%. Among them, **NVB**, suramin, aurintricarboxylic acid, and reactive blue 2 resulted the most potent inhibitors, but only **NVB** showed high specific POLθ inhibition in a ^32^P-based radiometric ATPase assay and, thus, further studies were focused on **NVB**.

**NVB** inhibited POLθ ATPase activity with an IC_50_ of 24 μM but did not significantly inhibit other six DNA repair-related proteins (HSP90AA1, TRIP13, helicase BLM, RAD51, SMARCAL1, and CHD1). Specificity of **NVB** for POLθ was also confirmed: (i) by using **NVB**-conjugated beads, which pulled down both purified POLθ ATPase domain (but not purified SMARCAL1, CHD1, BLM, or RAD51) and POLθ-GFP expressed in cells, and (ii) by a thermal shift assay, in which **NVB** was able to stabilize POLθ-GFP from cell lysate and to increase in a dose-dependent manner the stability of purified POLθ but not of BLM and MRE11.

**NVB** was also found to inhibit MMEJ activity in U2OS cells (GFP-based alternative end joining reporter assay), but it had little effect on HR activity (GFP-based direct repeat reporter assay). Analogously to POLθ depletion, gamma irradiation in the presence of **NVB** of U2OS cells slightly increased RAD51 foci and γH2AX foci.

*In vivo* studies were performed to determine the activity of **NVB** in killing HR-deficient tumor cells. **NVB** treatment significantly reduced BRCA1-deficent genetically engineered mouse model derived tumors and prolonged the overall survival of tumor-bearing mice. Moreover, **NVB** impaired the growth of FANCF-deficient TOV21G cancer cells (human ovarian cancer with reduced HR capacity) in a mouse xenograft study without affecting FANCF-complemented cells (TOV21G with *FANCF* cDNA). Results obtained *in vivo* were further confirmed by *in vitro* CSA, which showed that **NVB** significantly reduced the survival of *BRCA1*^*–/–*^ and *BRCA2*^*–/–*^ (*TP53*^*–/–*^) RPE1 cells, compared to isogenic WT cells. **NVB** exposure also induced apoptosis in *BRCA1*^*–/–*^ but not in *BRCA1*^*wt*^ cells in a dose-dependent manner, and chromosomal aberrations and radial chromosomes in *BRCA1*^*–/–*^ cells, confirming that **NVB** increased DNA damage.

Next, the authors demonstrated that POLθ was the major target of **NVB** in human cells by using *POLQ*^*–/–*^ RPE1 and U2OS cells, which showed higher tolerance to **NVB** compared to WT cells. Moreover, since **NVB** had been previously described as an inhibitor of HSP90 and TOP2, the authors demonstrated that **NVB** treatment at the concentration killing HR-deficient cells did not inhibit these enzymes, discarding the hypothesis that the cytotoxic effect of **NVB** was caused by off-target inhibition of HSP90 and TOP2.

The synergism between **NVB** and PARPi was also evaluated. In particular, **NVB** reduced the IC_50_ of **rucaparib** by more than 20-fold in *BRCA1*^*–/–*^ RPE1 cells and the IC_50_ of **olaparib** by more than 40-fold in HR-deficient TOV21G cells, while it was unable to sensitize BRCA-proficient WT cells to PARPi. This synergistic effect was further demonstrated *in vivo* by using nonobese diabetic SCID gamma mice bearing tumors of the HR-deficient DF83 ovarian cancer patient-derived xenograft model (PDX). Complete tumor regression was observed by administering a submaximal dose of **NVB** and **olaparib** in combination, without significant toxicity. Only a small degree of tumor growth inhibition and partial tumor regression were instead observed by using **olaparib** and **NVB** alone, respectively. Additionally, in order to verify whether POLθ inhibitors could be used to treat PARPi-resistant tumors, the PARPi-resistant PDX model DF59 not responsive to **olaparib** was used. **NVB** alone inhibited tumor growth, also observing a tumor regression when administered in combination with **olaparib**.

To understand the mechanism that allowed **NVB** to overcome PARPi resistance, the authors generated four **olaparib**-resistant clones from *BRCA1*^*–/–*^ RPE cells, showing multiple PARPi-resistance mechanisms. Clones showed: (i) fork stabilization, (ii) RAD51 foci restoration, (iii) decreased expression of REV7, and/or (iv) decreased expression of 53BP1. These data suggested a restoration of HR repair activity that could partly result from downregulation of Shieldin complex and subsequently of NHEJ repair. All the clones did not show BRCA1 re-expression and were sensitive to **NVB**, indicating that **NVB** could overcome multiple mechanisms of acquired PARPi-resistance. Depletion of POLθ in both clones and parental *BRCA1*^*–/–*^ cells led to reduced cell survival, confirming POLθ inhibition as the mechanism for **NVB** cytotoxicity.

The mechanism by which **NVB** kills cancer cells was investigated by measuring DNA resection at DSBs generated by restriction endonuclease AsiSI (induced by 4-hydroxytamoxifen) in U2OS cells by (q)PCR, in the presence or absence of **NVB**. Cells treated with **NVB** showed an amount of ssDNA around the DSB significantly higher compared to those treated with DMSO.

To test if **NVB** was able to induce nonfunctional RAD51 foci, the latter were evaluated in tumor cells from PDX model treated with PARPi and/or **NVB** (HR-deficient tumors with PARPi resistance). Results showed that **NVB** induced elevated DSB end resection and subsequent accumulation of ssDNA intermediates and nonfunctional RAD51 loading, suggesting that this could be the mechanism of cell killing by **NVB** in HR-deficient PARPi resistant tumor cells. Finally, the authors determined that POLθ expression levels correlated with cellular sensitivity to **NVB**, thus representing a predictive biomarker.

In 2022, D’Andrea and co-workers reported an interesting study in which they demonstrated that the simultaneous disruption of the two DNA repair pathways MMEJ, via POLθ inhibitor **NVB**, and NHEJ, via DNA-PK inhibitor **peposertib**, led to accumulation of toxic levels of DSB end resection and apoptosis-mediated cell death, resulting in synergistic SL.^121^ The synergistic action of the combined treatment was observed across multiple cancer cell lines, including HR-proficient and HR-deficient cells. Moreover, a combination of **NVB** with **peposertib** exhibited SL in *TP53*-deficient tumor cells, without the use of additional cytotoxic chemotherapy. In particular, *TP53*-deficient cell lines, organoid cultures and PDX models that were **peposertib**-resistant showed an increased sensitivity to **NVB**. Based on these results, the authors hypothesized that *TP53*-deficient cancer cells acquired resistance to DNA-PK inhibitor **peposertib** thorough a compensatory increase of POLθ expression and MMEJ pathway, of which they became dependent, thus showing sensitivity to **NVB**. Interestingly, *TP53*-deficient cancer cells treated with **NVB** recovered sensitivity to **peposertib**, strongly supporting the combination therapy based on NHEJ and MMEJ inhibitors in *TP53* mutant cancers.

## 3 Structural Analysis of Known POLθ Inhibitor Binding Sites

Atomic structures of full length POLθ protein have not been reported yet, although crystal structures of the POLθ-hel and POLθ-pol domains are available (Table 2).

**Table 2:** Summary of Available 3D Structures of POLθ Protein (Accessed on March 31, 2023)

PDB	POLθ domain	resolution	global symmetry	ligand	ref
5A9J	helicase	3.55 Å	monomer	no	(69)
5A9F	helicase	3.20 Å	tetramer	ADP	(69)
5AGA	helicase	2.90 Å	tetramer	AMP-PNP	(69)
ND^a^	helicase	3.27 Å	dimer	no	(122)
ND^a^	helicase	3.14 Å	dimer	**NVB**	(122)
4X0Q	polymerase	3.90 Å	monomer	ddGTP	(123)
4X0P	polymerase	3.91 Å	monomer	ddATP	(123)
6XBU	polymerase	3.29 Å	dimer	ddGTP	(70)
7ZUS	polymerase	2.26 Å	monomer	ddGTP	(98)
7ZX0	polymerase	2.99 Å	monomer	ddGTP and **10**	(98)
7ZX1	polymerase	2.83 Å	monomer	ddGTP and **ART588**	(98)
8E23	polymerase	2.59 Å	dimer	ddGTP and **14**	(97)
8E24	polymerase	2.34 Å	dimer	ddGTP and **15**	(97)

a ND = not disclosed.

Crystal structures of POLθ-hel (N-terminal) domain include protein residues ranging from 1 to 894 bound to either ADP (PDB 5A9F) or adenosine 5′-(β,γ-Imino)triphosphate (AMP-PNP, PDB 5AGA), as well as the apo form (PDB 5A9J).^69^ Recently, two additional cryo-EM structures of POLθ-hel were reported in the apo form and in complex with an inhibitor.^122^ Eight POLθ-pol (C-terminal) domain crystal structures are available and include residues spanning from 1823 to 2590, a primer:template (DNA:DNA or DNA:RNA) chain of nucleic acid and an incoming 2′,3′-dideoxynucleotide triphosphate (ddGTP or ddATP). It is worth noting that, in the first released structures, the incoming nucleotide was the only cocrystallized ligand (i.e., 4X0Q,^123^4X0P,^123^6XBU,^70^ and 7ZUS(98)), whereas the recently disclosed complexes (i.e., 7ZX0,^98^7ZX1,^98^8E23,^97^ and 8E24(97)) showed both a nucleotide and an allosteric inhibitor bound to POLθ-pol.

Despite the availability of POLθ domains crystal structures, no structure-based virtual screening campaigns have been reported to date, while the recently disclosed cocrystal structures of POLθ-pol domain have been used in the SBDD approach previously mentioned that led to the discovery of potent inhibitors (i.e., **ART812**(98) and **RP-6685**,^97^Figures 5 and 6) able to impair POLθ-pol activity by binding an allosteric pocket.

Apart from the latest experimental structures released, it should be noted that, in general, the structural studies conducted on POLθ were mainly targeted to investigate the biological activity and mechanistic aspects of the protein enzymatic function, and therefore the available information on the presence of druggable regions in the POLθ structure were very limited. Consequently, the 3D structures of both polymerase and helicase are still poorly explored from a drug discovery perspective. A discussion of POLθ’s structure and mechanisms of action is beyond the scope of this review, and readers are directed to recent reviews^26,124−126^ for a comprehensive discussion on this topic. However, the literature data were analyzed focusing on the well-characterized inhibitors binding pockets that could be explored within drug discovery programs based on SBDD approach. A summary of this analysis is shown in Table 3, in which we report the residues composing the most interesting regions to be explored for each POLθ domain.

**Table 3:** Residues Composing the Described POLθ Protein Pockets/Surfaces

pocket/protein surface	residues
Helicase Domain	
NVB-binding site	145, 146, 147, 148, 151, 175, 220, 223, 422, 423, 749, 750, 753
Polymerase Domain	
active site	2241, 2254, 2330, 2331, 2332, 2333, 2334, 2335, 2357, 2359, 2379, 2380, 2382, 2383, 2384, 2387, 2388, 2391, 2470, 2474, 2538, 2540, 2541, 2575, 2603
allosteric site	2348, 2362, 2365, 2385, 2386, 2389, 2402, 2412, 2415, 2416, 2420, 2419

### 3.1 Targeting POLθ-pol Pockets

The overall organization of the POLθ-pol domain is similar to that of Taq DNA polymerase, where the exonuclease, thumb, and finger subdomains are arranged around a right-hand palm subdomain. Additionally, in the POLθ-pol structure, five unique and specific insertion loops are present.^123^ These sequence inserts represent protein sequences distinct from other helicase or polymerase structures and can be considered unique features of POLθ.^69^ The palm subdomain hosts the strictly conserved catalytic aspartate and glutamate residues (Asp2330, Asp2540, and Glu2541) responsible for the coordination of a divalent Ca^2+^ ion essential for the enzymatic activity of the protein.^123^

**Figure 12:**
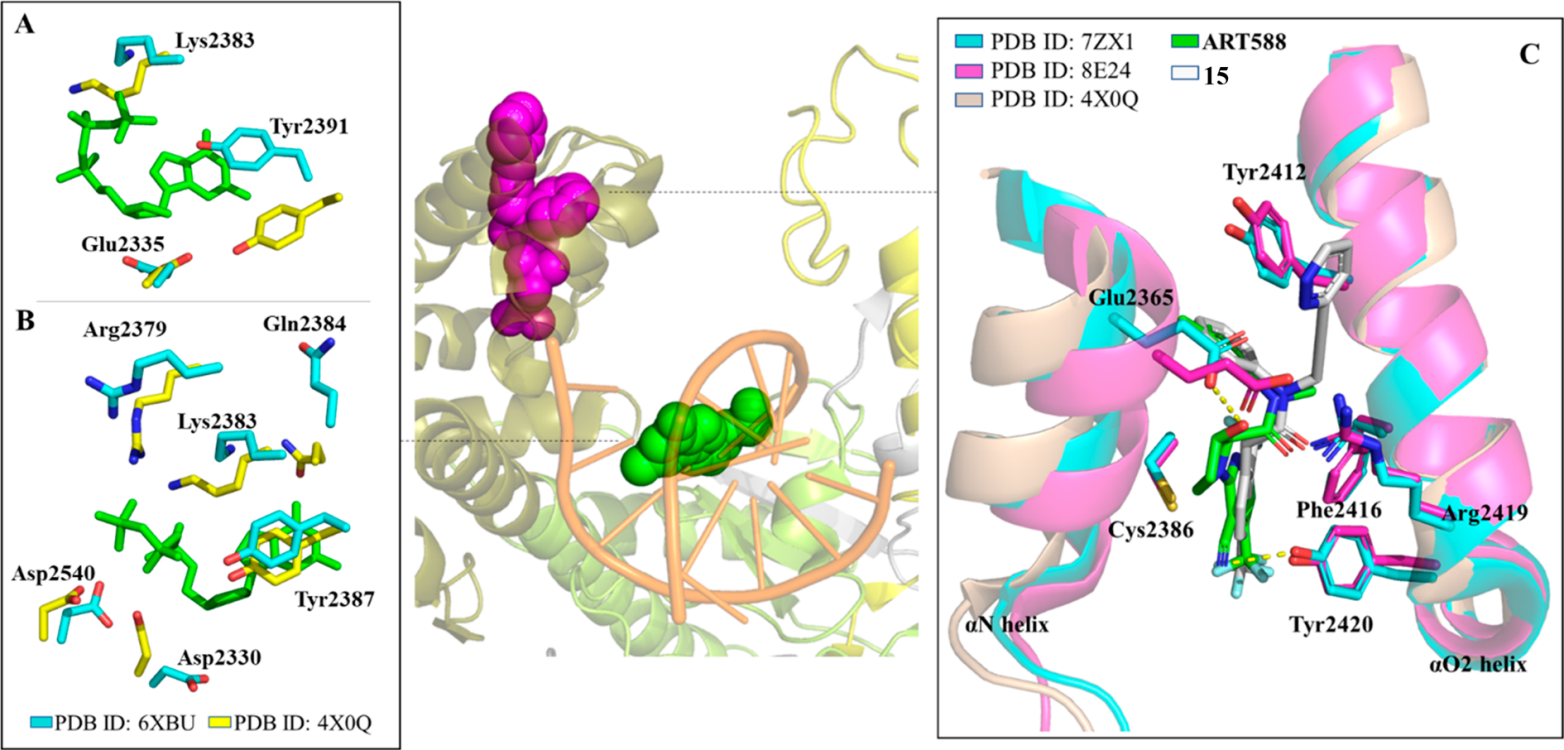
Overview of the POLθ-pol domain (PDB 8E24(97)) and relative location of the active and allosteric sites. The cocrystallized ligands ddGTP (green CPK spheres) and **15** (magenta CPK spheres) bound the active site and allosteric site, respectively. (A) Protein residues interacting with ddGTP in the POLθ-pol:DNA–RNA complex (cyan; PDB 6XBU(70)) compared to the same residues in the POLθ-pol:DNA–DNA complex (yellow; PDB 4X0Q(123)). (B) Protein residues interacting with ddGTP in the POLθ-pol:DNA–DNA complex (yellow; PDB 4X0Q) compared to the same residues in the POLθ-pol:DNA–RNA complex (cyan; PDB 6XBU). (C) Close-up view on the allosteric binding site of POLθ-pol bound to the cocrystallized inhibitors **15** (gray) and **ART588** (green). The relative position of the αN and αO2 helices was represented both in the presence (PDB 7ZX1(98) and 8E24(97)) and in absence (PDB 4X0Q) of bound inhibitors..

The POLθ-pol domain contains two well characterized ligand binding sites, named the active site and the allosteric site (Figure 12). Most of the approved drugs targeting polymerases block the enzymatic activity by binding to the active site of the corresponding targeted protein, making this pocket an appealing druggable site also in the POLθ-pol domain. Indeed, the first PA disclosing POLθ inhibitors back in 2017 (WO2018/035410A1)^99^ reported synthetic xDNA (Figure 2) nucleotides and analogues that were efficiently used by POLθ as substrates, thus inhibiting the POLθ DNA synthesis activity by targeting the active site of POLθ-pol domain. This pocket hosts the three well-conserved catalytic aspartate and glutamate residues (Asp2330, Asp2540, and Glu2541) and can accommodate one nascent DNA chain, an RNA–DNA template chain, and an incoming nucleotide. Interestingly, depending on the template chain used (i.e., DNA or RNA), the structural organization of the active site residues can change, leading to a modification of the intermolecular interactions established by the incoming nucleotide and POLθ-pol residues. As an example, Pomerantz and co-workers reported the importance of the Tyr2391 in stabilizing the ddGTP in the PDB 6XBU structure, in which the POLθ-pol domain is bound to an RNA template molecule (Figure 12A).^70^ However, the same residue did not establish any interaction in the PDB crystal structure 4X0Q,^123^ where a DNA template molecule is present (Figure 12B). This active site plasticity needs to be considered in rational drug discovery approaches aimed at identifying novel small molecule binders of the POLθ-pol domain.

Regarding the recently disclosed allosteric site on the POLθ-pol domain,^97,98^ this pocket is flanked by six α-helices and located in the finger subdomain opposite to the incoming ddGTP (Figure 12). As already mentioned, the discovery and characterization of this site (Table 3) took place thanks to cocrystallization studies conducted on hit compounds identified using a HTS approach on the POLθ-pol domain. The allosteric pocket, created by the movement of Tyr2412 and Phe2416, is mainly composed by hydrophobic residues and presents two exit paths separated by the side chains of Arg2419 and Glu2365. Interestingly, it was demonstrated that the occupation of these paths can enhance the affinity of the inhibitors to the pocket. Indeed, the optimization of compounds **14** and **10** to compounds **15** and **ART558**, respectively, led to the establishment of a new interaction with Glu2365 that dramatically improved the inhibitor potency. However, while **ART588** performed a H-bond contact with the Glu2365 side chain, the presence of compound **15** in the allosteric binding site prompted the formation of a salt bridge between Glu2365 and Arg2419, which was absent in the apo protein. From the structural point of view, this intramolecular interaction was allowed thanks to the movement of the helix αN (Figure 12C).

### 3.2 Targeting POLθ-hel Pockets

The POLθ-hel domain is a member of the superfamily 2 (SF2) helicases. The domain comprises five subdomains (D1–5), of which two are the core helicase domains and three are closely associated globular domains. The RecA-like domains D1 and D2 (residues 1–289 and 290–513, respectively) share a prototypical fold similar to *E. coli* RecA and contain the core machinery required for helicase activity, including ssDNA-binding motifs, the nucleotide-binding site, and all of the core helicase motifs that are conserved across SF2-family helicases.^69^

A recent study described the cryo-EM structure of POLθ-hel domain in complex with inhibitor NVB (Figure 11).^122^ Initially, biochemical assays were performed to determine the mechanism of action of NVB. In particular, ATP and ssDNA competition assays performed with different concentration of NVB indicated a noncompetitive and competitive inhibition with respect to ATP and ssDNA, respectively. Then, cryo-EM structure of the POLθ-hel domain in the apo state and in complex with NVB were determined, showing a high similarity (root-mean-square deviation of 0.94 Å). Of note, POLθ-hel domain in the cryo-EM structures forms a homodimer rather than a tetramer as observed in the previously reported crystal structures.^69^

The NVB-POLθ-hel complex structure (PDB not disclosed) revealed that the NVB binding site is distinct from the canonical NVB binding site within the GyrB subunit of *Staphylococcus aureus* DNA gyrase, which is located next to the ATP binding site.^127^ Of note, the binding of NVB to a noncanonical allosteric site was already reported for lipopolysaccharide-transport proteins,^128^ while binding to the canonical binding site was reported for other proteins, such as topoisomerase IV (ParE)^129^ and HSP90.^130^

**Figure 13:**
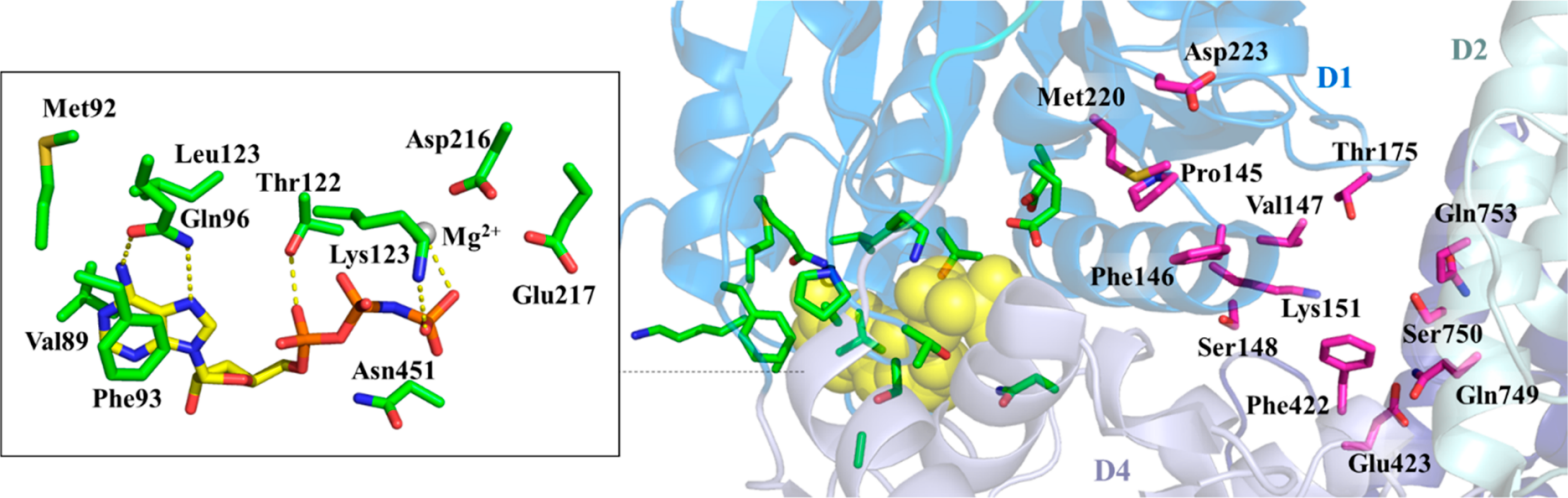
Overview of the POLθ-hel domain (PDB 5AGA(69)) with the ATP-binding site and NVB-binding site residues highlighted in green and magenta, respectively. The cocrystallized ligand AMP-PNP is represented in yellow. Details on the intermolecular interaction involving AMP-PNP and POLθ-hel are also illustrated.

In the POLθ-hel domain, the NVB binding pocket is a cleft nestled in the central tunnel of POLθ-hel structure, and the bound ligand is in close contact with D1, D2, and D4 subdomains (Figure 13). The binding of NVB at this site did not affect the overall organization of POLθ-hel but increased its stability in a dose-dependent manner. The analysis of the protein–ligand interactions revealed the presence of strong and favorable intermolecular contacts. Specifically, the coumarin system established a complex network of interactions with Phe422 (D2 subdomain, aromatic contacts), Val147, and Ser148 (D1 subdomain, H-bond contacts), and Val147 and Gln753 (D1 and D4 subdomains, respectively, hydrophobic contacts). The D1 domain residues Pro145, Phe146, Met220, and Asp 223 stabilized the apolar hydroxyl benzoate isopentyl moiety by performing hydrophobic interactions. Additionally, polar contacts were described between the novobiose sugar and the D1 residues Leu151 and Thr175, the D2 residue Glu423, and the D4 residues Gln749 and Ser750.

The extensive positively charged surface inside and outside the central tunnels hosting the NVB binding site (especially the surface from D2) suggested a potential nucleic acid binding interface and that NVB might compete with ssDNA for protein binding. To test this hypothesis, the NVB-POLθ-hel structure was superposed to the Hel308–DNA complex structure (PDB 2P6R(131)) because the sequence feature of POLθ-hel domain is closely related to HELQ/Hel308-type helicases, as well as RecQ-type helicases. Overall, the structures are similar, but differential conformational changes happen in different domains, with D4 exhibiting the most extensive global conformational changes. Moreover, in the DNA-bound structure, a larger space in the central tunnel was revealed, which implies conformational changes when ssDNA and DNA duplex bind to the helicase domain. Focusing on the NVB binding pose, the inhibitor overlaps with 3′ overhang ssDNA and, in particular, vertically wedges between the overhang ssDNA’s third and fourth unpaired bases. These data support that NVB competitively impairs ssDNA binding and translocation.

Other SF2 family helicases have been targeted by small molecules in the context of SL, such as the homologue Brahma-related gene 1 (BRG1, also known as SMARCA4) and the Brahma homologue (BRM, also known as SMARCA1), which belong to the Snf2-subfamily helicases, and the human Bloom syndrome protein (BLM), which is a RecQ helicase. Notably, inhibitors of these helicases showed a different ligand binding pocket and mechanism of action with respect to the POLθ-hel inhibitor NVB. Specifically, BRG1 and BRM are mutually exclusive DNA-dependent ATPases of the large ATP-dependent SWI/SNF chromatin-remodeling complexes, which are involved in transcriptional regulation of gene expression. BRM is a critical SL target in BRG1-deficient cells.^132^ On the other hand, BLM is involved in the dissolution of complex DNA structures and repair intermediates, and it was found to be a promising SL target in a range of cancers with defects in the DNA damage response.^133^

In 2018, a class of potent dual urea-based inhibitors of BRM and BRG1 ATPase activity was reported.^134^ Mechanistic studies showed that compounds are partially competitive with ADP (SPR assay using BRM) with a stoichiometric binding (SPR assay using BRM and isothermal titration calorimetry using BRG1). Accordingly, cocrystal structure of compounds with a fusion protein consisting of maltose-binding protein and the N-RecA lobe (705–960) of the BRM ATPase domain (PDBs: 6EG3 and 6EG2) confirmed that compounds bind an allosteric pocket in the vicinity of the ATP binding site, with the urea moiety trapping the catalytic Glu852.

In 2011, BLM inhibitors were cocrystallized with the target protein.^135^ Initially, mechanistic studies indicated that a substituted benzamide compound does not interfere with BLM ATP-binding site (unwinding assay) but has a noncompetitive mode of inhibition with respect to ATP (ATP-turnover experiments). Co-crystallization of BLM (the conformationally flexible Winged Helix domain was replaced by a short poly glycine/serine linker connecting the Zinc-binding domain to the helicase and RNase C-terminal domain) with the benzamide compound, ADP/magnesium cofactor, and ssDNA-15 mer (PDB 7AUD) confirmed that the inhibitor binds to an allosteric pocket. This small binding site is located on the protein face opposite to that binding the nucleotide and integrates amino acid side chains from both subdomains of the helicase core and the Zn-binding domain. The authors proposed that engagement of ssDNA by BLM led the protein aromatic-rich loop to acquire a particular conformation, permitting the binding of the inhibitor to the allosteric pocket, occupied and occluded by Trp803 in absence of ssDNA. The ligand–protein interaction acts to “trap” or stall the protein into a DNA-bound translocation intermediate, thus impairing the conformational changes required for progression to the successive step of the catalytic cycle. Nevertheless, further kinetic studies to determine if benzamide compound has a passive or induced binding mode are required.

## 4 Conclusions

Targeted therapies are considered among the most advanced anticancer strategies, moving from “one-size-fits-all” treatment to biomarker-driven therapy, which relies on the patient-specific characteristics. In the framework of precision medicine, the use of inhibitors of DSB repair proteins, which induces cell death based on the SL interactions, is a promising anticancer therapeutic approach.

Since the success of PARPi in targeting *BRCA*-mutated cancer cells, there has been a growing interest in identifying DDR factors as potential SL targets, even more strengthened by the increased onset of PARPi resistance. Based on the observation that POLθ is overexpressed and becomes essential in HR-deficient tumors, increasing attention has been paid to POLθ as a novel SL target. The identification of POLθ inhibitors is an emerging field, in which a few pharmaceutical companies are the main players. Nevertheless, the drug discovery efforts reported so far and herein described have already provided exciting results, giving an outstanding contribution not only to the discovery of POLθ inhibitors and the demonstration of their potential therapeutic effectiveness, but also to expanding knowledge about the role of POLθ in different cancer frameworks, including some forms of drug-resistance.

First, inhibition of both the POLθ-pol and POLθ-hel domains has been proved to be feasible with small molecules. Specifically, the former has been the most pursued, leading to the identification of compounds endowed with anti-POLθ activity with potency in the low nanomolar range, while only a few POLθ-hel inhibitors have been reported, of which antibiotic **NVB** was the best characterized. Focusing on POLθ-pol inhibitors, several chemical series have been reported by different pharmaceutical companies, and all those described to date, with the exception of nucleotide analogues and urea derivatives, have some chemical features in common. Moreover, cocrystal studies performed on two of them (**ART** and **RP** inhibitors) showed that they bind to the same allosteric pocket, leading us to hypothesize that all the reported compounds act as allosteric inhibitors targeting the same pocket.

Second, very important insights came from studies performed on POLθ-pol inhibitor **ART558** and POLθ-hel inhibitor **NVB**. Initial characterization of these compounds included studies to understand their mechanism of action and target engagement, as well as the assessment of their selectivity against other targets. They were then used as tools to increase the knowledge on POLθ biology as well as investigate the relevance of targeting POLθ in cancer. In addition to providing the first proof that the SL effect between POLθ inhibition and *BRCA*-deficiency, initially identified using genetic assays, could be reproduced by selective small molecule, these studies showed that POLθ inhibitors were able to (i) elicit SL with *BRCA* genes through inhibition of at least one of the two POLθ activities, (ii) show selective killing of HR-deficient cells over wild-type cells, and (iii) potentiate the cytotoxic effect of PARPi in HR-deficient tumor cells. Most importantly, POLθ inhibitors could be exploited to target some forms of drug-resistance, such as PARPi resistance caused by 53BP1/Shieldin defects in *BRCA1* mutant cancers and DNA-PK inhibitors resistance in *TP53* mutant cancers. These findings led to recognition of new genes that are SL partners with POLθ inhibitors (alone or in combination with other drugs), not only expanding the potential clinical use of inhibitors, but also identifying new predictive biomarkers that are essential in DDR inhibitor clinical trial design to determine whether a tumor is likely to be sensitive or resistant to targeted therapy.^59^ In particular, loss of 53BP1/Shieldin or NHEJ, as well as high *POLQ* expression are all predictive biomarkers for POLθ inhibitors responsiveness. Moreover, *BRCA1*- or *TP53*-deficiency could serve as predictive biomarker of the sensitivity of a tumor to POLθ inhibitors in combination with PARPi and DNA-PK inhibitors, respectively.

The work done on both **ART558** and **NVB** qualifies them as important tools to explore the biology of POLθ. The POLθ-pol inhibitor **ART558** has been more extensively characterized than the POLθ-hel inhibitor **NVB**, as it was tested on many oncology-relevant targets besides other human DNA polymerases. Therefore, **ART558** has been added to the chemical probes listed in the Chemical Probe Portal^136^ (https://www.chemicalprobes.org) and is currently in the process of review by the Scientific Expert Review Panel (SERP) to assign it a rating. Additional *in vitro*/*in vivo* characterization of **ART558** will certainly favor its qualification as a high-quality chemical probe.^137^ As such, it will represent an important asset in the efforts to expand the knowledge on the biology of POLθ.

Third, the search for POLθ inhibitors has already moved past the discovery phase and has identified drug candidates. In 2022, POLθ-pol inhibitor **ART4215** has started a Phase 2 study in combination with PARPi **talazoparib** for the treatment of *BRCA*-deficient breast cancer.^119^ Moreover, a potential first-in-class POLθ-hel development candidate is under evaluation in ongoing IND-enabling studies in combination with **niraparib** to treat *BRCA*-mutated or other HR-impaired cancer cells.

In conclusion, this review furnishes a snapshot of POLθ inhibitor discovery and development, highlighting the potential that POLθ inhibitors have in targeted anticancer therapy. Moreover, we provide our own additional insights that could be exploited to identify new POLθ inhibitor chemotypes. We propose a plausible 3D ligand-based pharmacophore model for POLθ-pol inhibitors that could be used to support LBDD approaches. In this context, we trust that this review may represent a comprehensive source of chemical and biological information on POLθ-pol inhibitors that could be exploited for the development of more advanced models using state-of-the-art machine learning algorithms, with the final aim of identifying new POLθ inhibitor chemotypes.

## Supporting Information Available

Figures reporting a detailed description of structural modification performed on compounds **1**–**16** and **22**–**26** and pharmacophore modeling details (PDF)

## Abbreviations Used

alt-EJ: alternative end joining
ATM: ataxia-telangiectasia mutated
ATR: ataxia-telangiectasia and Rad3 related
BER: base excision repair
BLM: bloom syndrome protein
BRG: Brahma-related gene 1
BRM: Brahma homologue
CSA: clonogenic survival assay
CTG: cell titer glow
DDR: DNA damage repair
DNA-PK: DNA-dependent protein kinase
DNA-PKcs: DNA-PK catalytic subunit
DSBs: DNA double-strand breaks
DSF: differential scanning fluorimetry
dxNMPs: expanded-size deoxyribonucleoside monophosphates
dxNTPs: expanded-size deoxyribonucleoside triphosphates
GSK: GlaxoSmithKline
HR: homologous recombination
Ku: Ku70–Ku80
MMEJ: microhomology-mediated end-joining
NHEJ: nonhomologous end-joining
MRN: MRE11, RAD50, and NBS
NVB: novobiocin
PARP: poly(ADP-ribose) polymerase
PARPi: PARP inhibitors
PAs: patent applications
PDX: patient-derived xenograft
PEA: primer extension assay
POLθ: polymerase theta
POLθ-hel: POLθ helicase
POLθ-pol: POLθ polymerase
PPi: inorganic pyrophosphate
RPA: replication protein A
SF2: superfamily 2
SL: synthetic lethality
SPR: surface plasmon resonance
SSA: single-strand annealing
ssDNA: single-stranded DNA
TMEJ: theta-mediated end-joining
xDNA: expanded-size DNA
