# Supplementary material for: Small Molecules Targeting DNA Polymerase Theta (POLθ) as Promising Synthetic Lethal Agents for Precision Cancer Therapy
Source: J Med Chem. 2023 May 3;66(10):6498–522. doi: 10.1021/acs.jmedchem.2c02101 (PMC10226047; doi:10.1021/acs.jmedchem.2c02101)
Supplement: Supplementary file 1 — jm2c02101_si_001.pdf [file jm2c02101_si_001.pdf]

Supporting Information

Small Molecules Targeting DNA Polymerase Theta (Polθ) as Promising Synthetic Lethal Agents for Precision Cancer Therapy

Maria Chiara Pismataro,[1,#] Andrea Astolfi,[1,#] Maria Letizia Barreca,[1] Martina Pacetti,[1] Silvia Schenone,[2] Tiziano Bandiera,[3] Anna Carbone[2,] and Serena Massari[1,*]*

[1] Department of Pharmaceutical Sciences, University of Perugia, Via del Liceo 1, 06123 Perugia, Italy

[2] Department of Pharmacy, University of Genoa, Viale Benedetto XV 3, 16132 Genoa, Italy

[3] D3 Pharmachemistry, Istituto Italiano di Tecnologia, Via Morego 30, 16163 Genova, Italy.

Corresponding Authors

Serena Massari - Department of Pharmaceutical Sciences, University of Perugia, Via del Liceo 1, 06123 Perugia, Italy; https://orcid.org/0000-0002-9992-6318; e-mail: serena.massari@unipg.it

Anna Carbone - Department of Pharmacy, University of Genoa, Viale Benedetto XV 3, 16132 Genoa, Italy; https://orcid.org/0000-0002-6767-2376; e-mail: anna.carbone1@unige.it

Table of Contents

Figures S1-S15. Figures reporting a detailed description of structural modification performed on compounds **1-16** and **22-26.** Pag. S2

Pharmacophore Modelling. Pag. S14

Table S1. Features composing the 3D-pharmacophore model and their distances. Pag. S15

References. Pag. S15

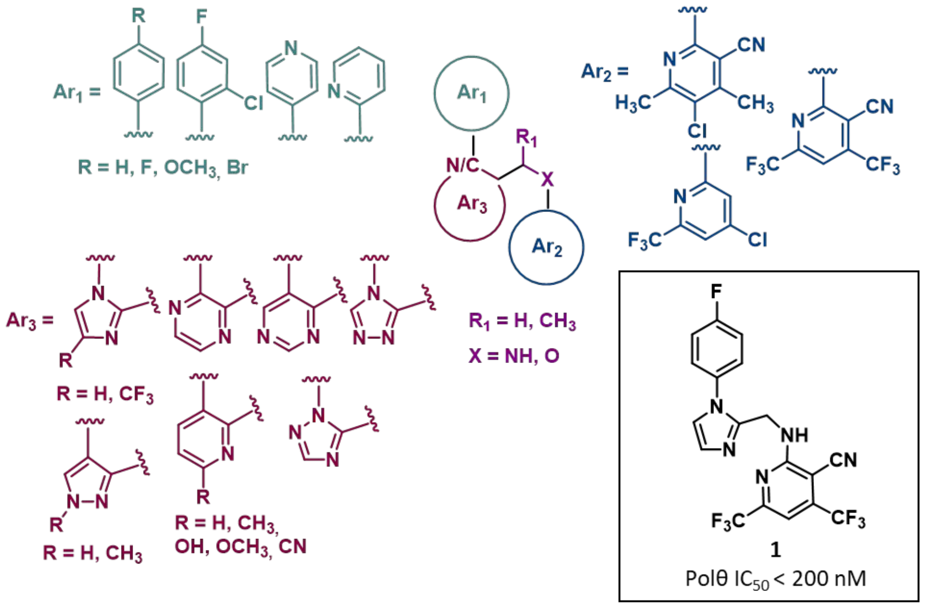

Figure S1. Structure of **heteroarylmethylene derivatives** as Polθ-pol inhibitors reported in PA WO2020/160213A1 by Ideaya.[1] The IC_{50} value represents the compound concentration that reduces by 50% the Polθ-pol activity as measured by PEA. Phenyl or nitrogen-based heterocycles were exploited as Ar_1 and Ar_2. The 4-fluorophenyl, 4,6-bis(trifluoromethyl)pyridine-3-carbonitrile and imidazole were the most common moieties as Ar_1, Ar_2 and Ar_3, respectively. In most of the compounds, Ar_2 and Ar_3 were linked by an unsubstituted methylene bonded to a nitrogen atom (**X**), while Ar_1 and Ar_3 were directly linked to each other.

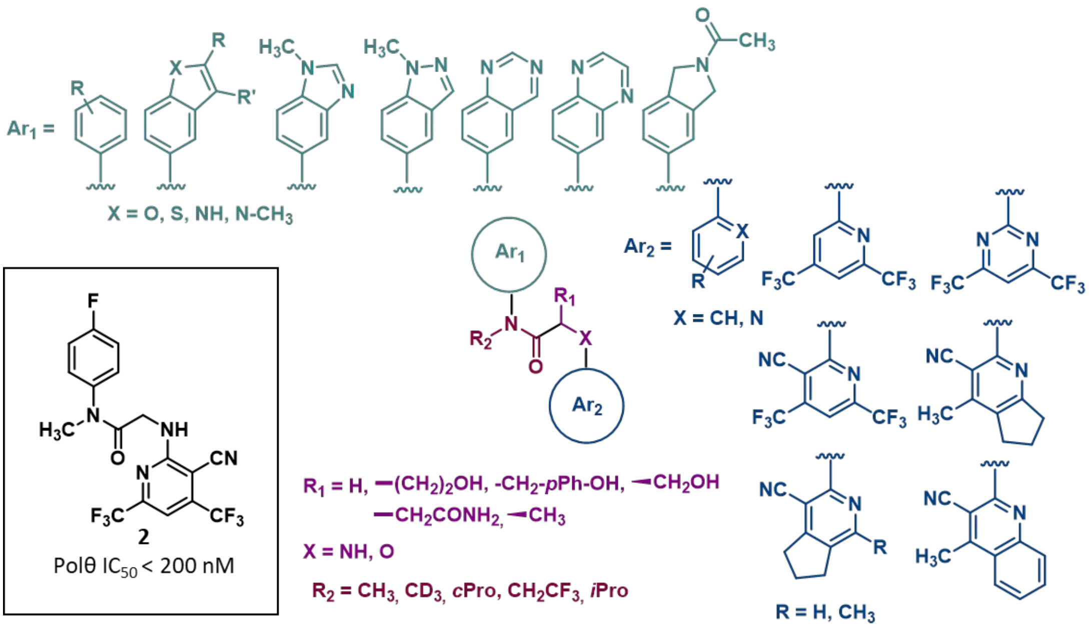

Figure S2. Structure of **acetamido derivatives** as Polθ-pol inhibitors reported in PA WO2020/160134A1 by Ideaya.[2] Due to the high number of possible substituents, some of them (R

and R') were omitted. The IC_{50} value represents the compound concentration that reduces by 50% the Polθ-pol activity as measured by PEA. Phenyl or nitrogen-based heterocycles were exploited as Ar_1 and Ar_2: the 4-fluorophenyl and 4,6-bis(trifluoromethyl)pyridine-3-carbonitrile were the most common groups, respectively, although other (hetero)cycles were investigated. Most compounds had a nitrogen atom as heteroatom and an unsubstituted methylene unit, although in five compounds different aliphatic substituents (R_1) were present. Various small aliphatic moieties were exploited as substituents (R_2) of the tertiary amide.

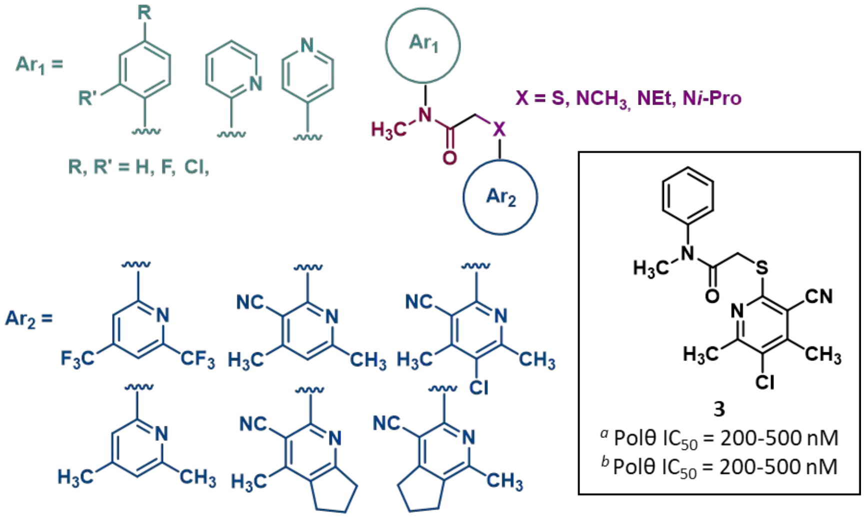

Figure S3. Structure of **acetamido-amino** and **acetamido-sulfur derivatives** as Polθ-pol inhibitors reported in PA WO2022/026548Al by Ideaya.[3] The IC_{50} value represents the compound concentration that reduces by 50% the Polθ-pol activity as measured by [a] PEA and [b] PPi assay. The *p*-fluorophenyl moiety and an unsubstituted phenyl ring were the most common Ar_1 groups. The amide nitrogen was substituted by a methyl moiety, while only in one compound the amidic Me-N-Ar_1 was part of a tetrahydroquinoline ring. The heteroatom within the N-C-C-X linker was frequently a sulfur atom, while only in three compounds it was a substituted nitrogen.

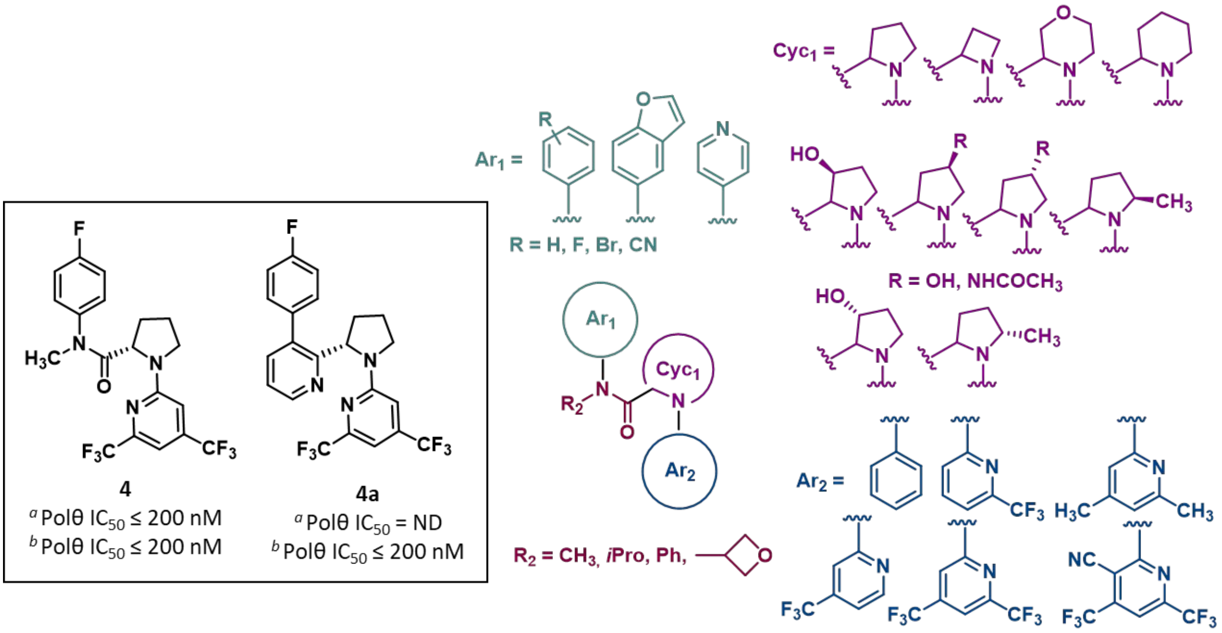

Figure S4. Structure of **cyclic acetamido derivatives** as Polθ-pol inhibitors reported in PA WO2022/026565A1 by Ideaya.[4] The IC_{50} value represents the compound concentration that reduces by 50% the Polθ-pol activity as measured by [a] PEA and [b] PPi assay. In most of the compounds, Cyc_1 was a pyrrolidine, Ar_1 was a *p*-fluorophenyl ring, Ar_2 was a 4,6-bis(trifluoromethyl)pyridine, and the amidic nitrogen was substituted by a methyl group. In three compounds, the amide link was replaced by a pyridine ring, as exemplified by compound **4a**.

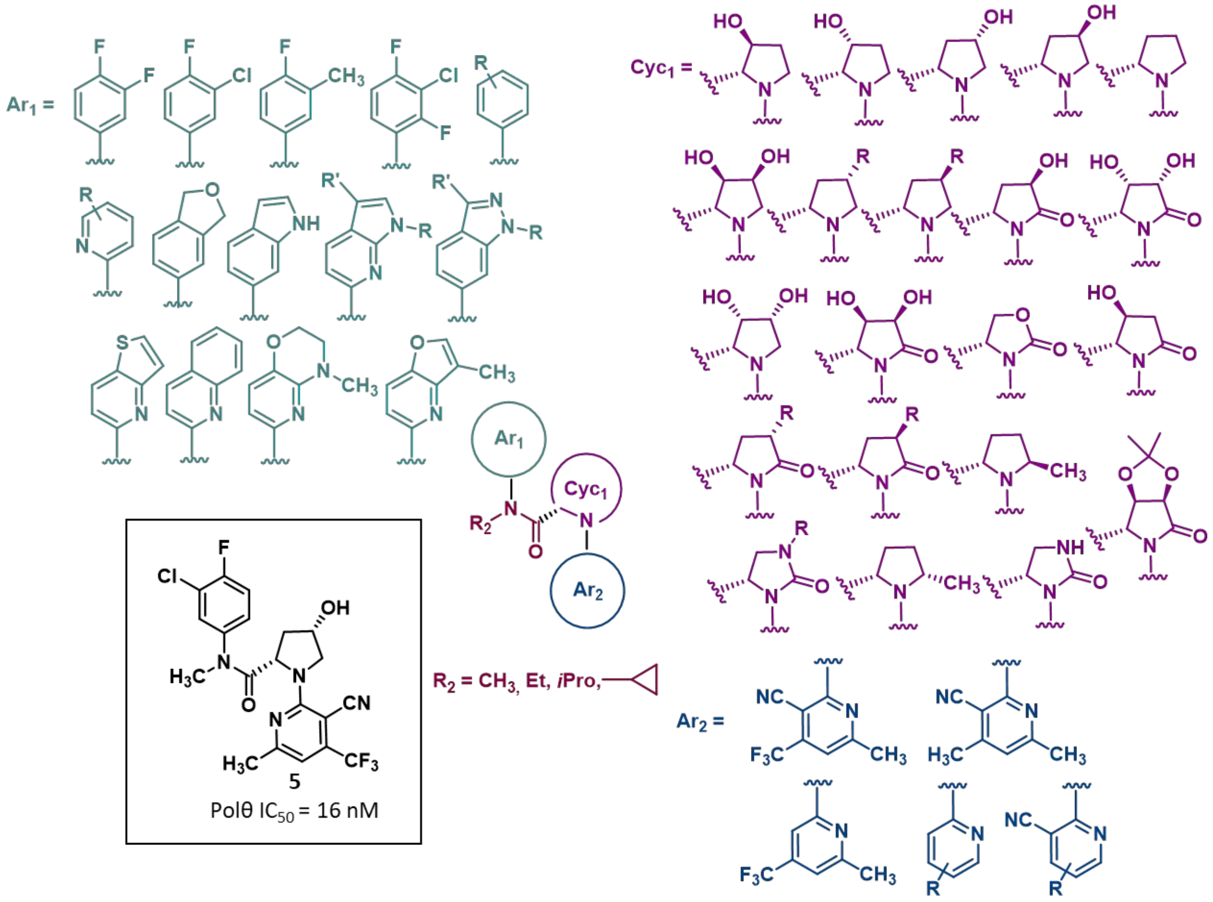

Figure S5. Structure of **heterocyclic amide derivatives** as Polθ-pol inhibitors reported in PA WO2021/028643A1 by Artios.[5] Due to the high number of substituents, some of them (R and R’) were omitted. The IC_{50} value represents the compound concentration that reduces by 50% the Polθ-pol activity as measured by PEA. Oxazolidin-2-one, 1-substitued imidazolidin-2-one, and 3-substituted pyrrolidine were the most frequent Cyc_1 heterocycles, all characterized by (*S*)-stereochemistry at the carbon atom bearing the amide group. Ar_1 was a bicyclic ring or a substituted phenyl/pyridine ring, of which a 3-chloro-4-fluorophenyl, 3-methylphenyl, 3-chloro-2,4-difluorophenyl, and 3,4-difluorophenyl were the most frequent groups. Ar_2 was a pyridine ring decorated with different substituents, such as a carbonitrile, a trifluoromethyl and/or a methyl, in most of the compounds.

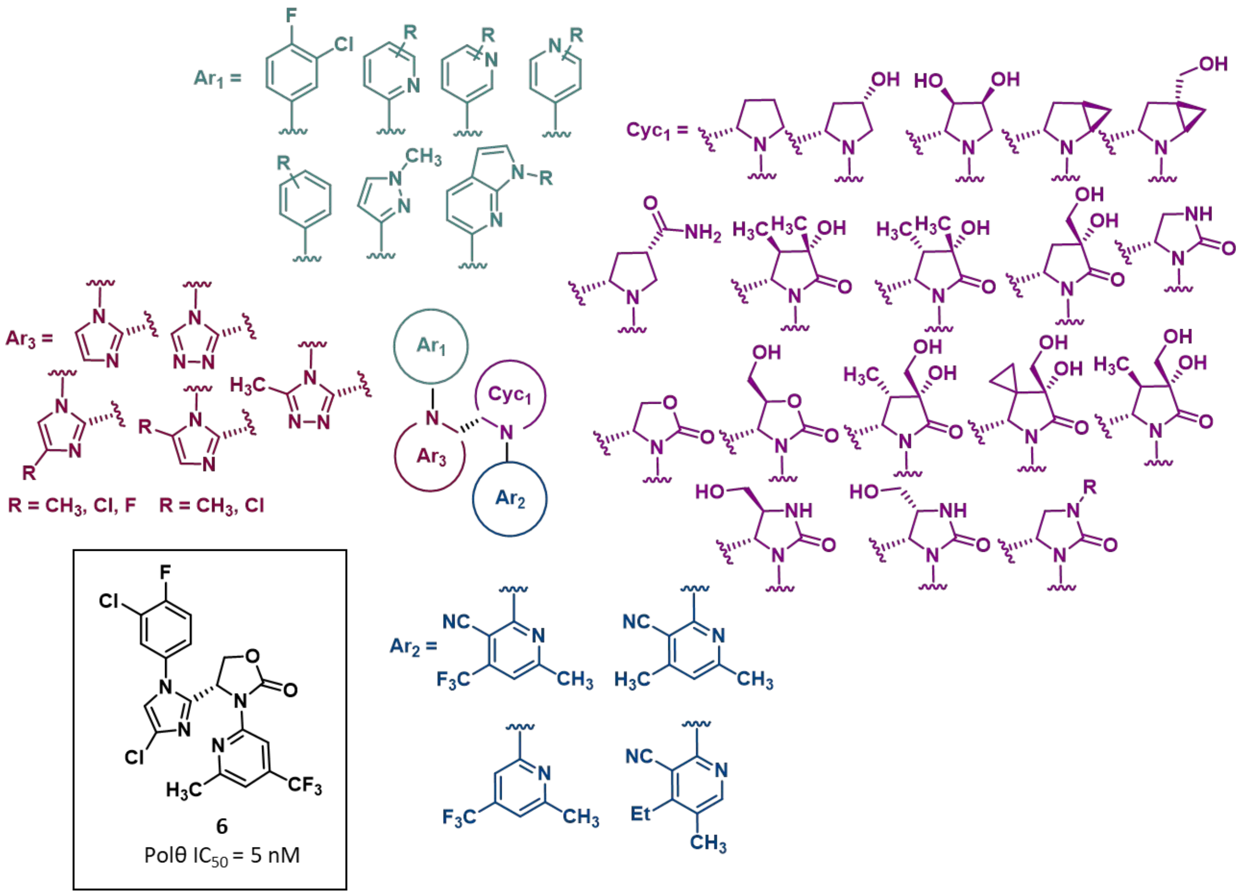

Figure S6. Structure of **heterocyclic derivatives** as Polθ-pol inhibitors reported in PA WO2021/123785A1 by Artios.[6] Due to the high number of substituents (R), some of them was omitted. The IC_{50} value represents the compound concentration that reduces by 50% the Polθ-pol activity as measured by PEA. In these compounds, all characterized by (*S*)-stereochemistry at the C-2 carbon atom of the pyrrolidine ring, the most frequent moieties were: i) a halo-substituted phenyl ring as Ar_1, such as the 3-chloro-4-fluorophenyl; ii) a substituted pyridine as Ar_2, such as the 6-methyl-4-(trifluoromethyl)nicotinonitrile and the 2-methyl-4-(trifluoromethyl)pyridine; iii) a nitrogen-based heterocycle as Cyc_1, such as the (3S)-pyrrolidin-3-ol or a pyrrolidine; and iv) a nitrogen-based heteroaromatic ring as Ar_3, such as an imidazole or a 1,2,4-triazole.

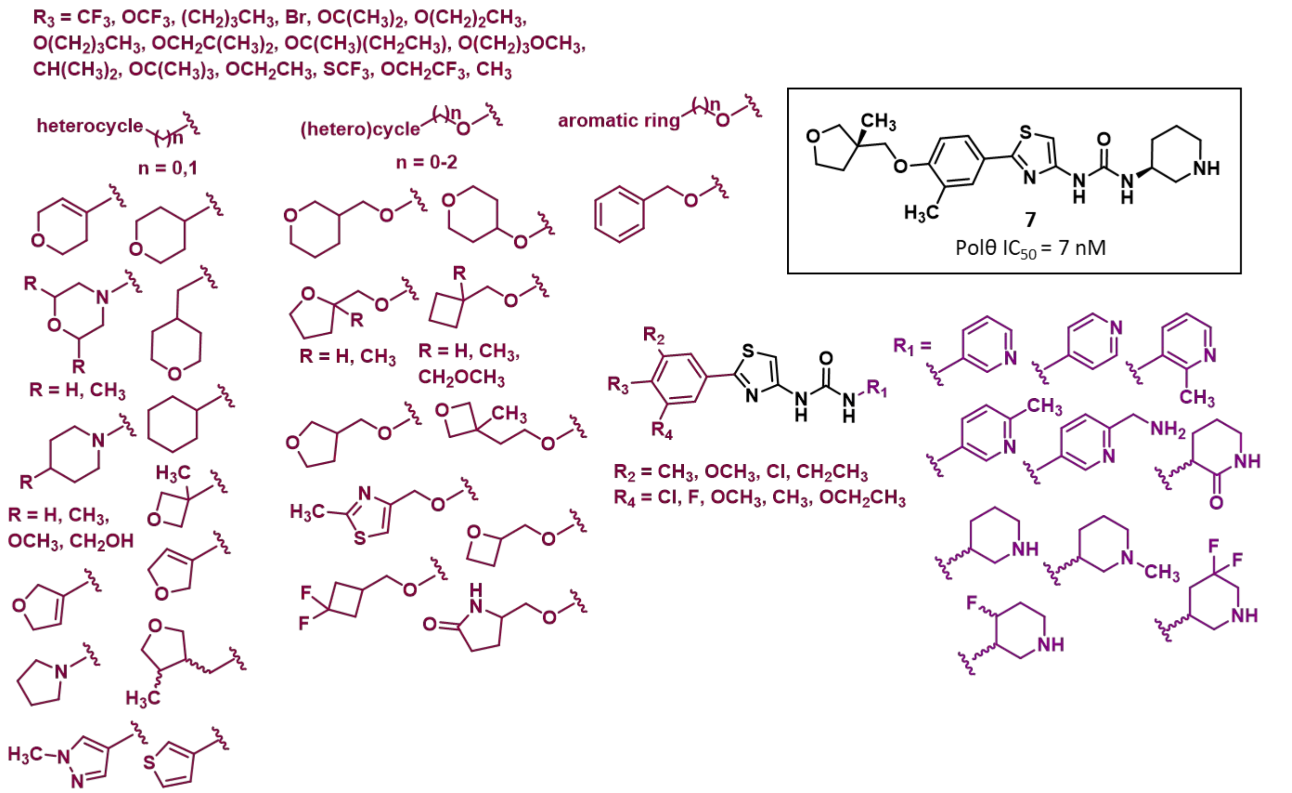

Figure S7. Structure of **thiazoleurea derivatives** as Polθ-pol inhibitors reported in PA WO2020/030924A1 by Artios.[7] Due to the high number of R_3 substituents, only some representative examples were reported. The IC_{50} value represents the compound concentration that reduces by 50% the Polθ-pol activity as measured by PEA. An unsubstituted piperidine was the most investigated R_1 substituent, followed by a 3-pyridinyl ring. Among the substituents of the phenyl ring (R_2, R_3 and R_4): i) a methyl group was the most frequent R_2 substituent; ii) R_3 was the most investigated by studying a number of different aliphatic and aromatic groups, of which the 3,6-dihydro-2*H*-pyran was the most frequent; and iii) the methyl and methoxy groups were the most used R_4 substituents.

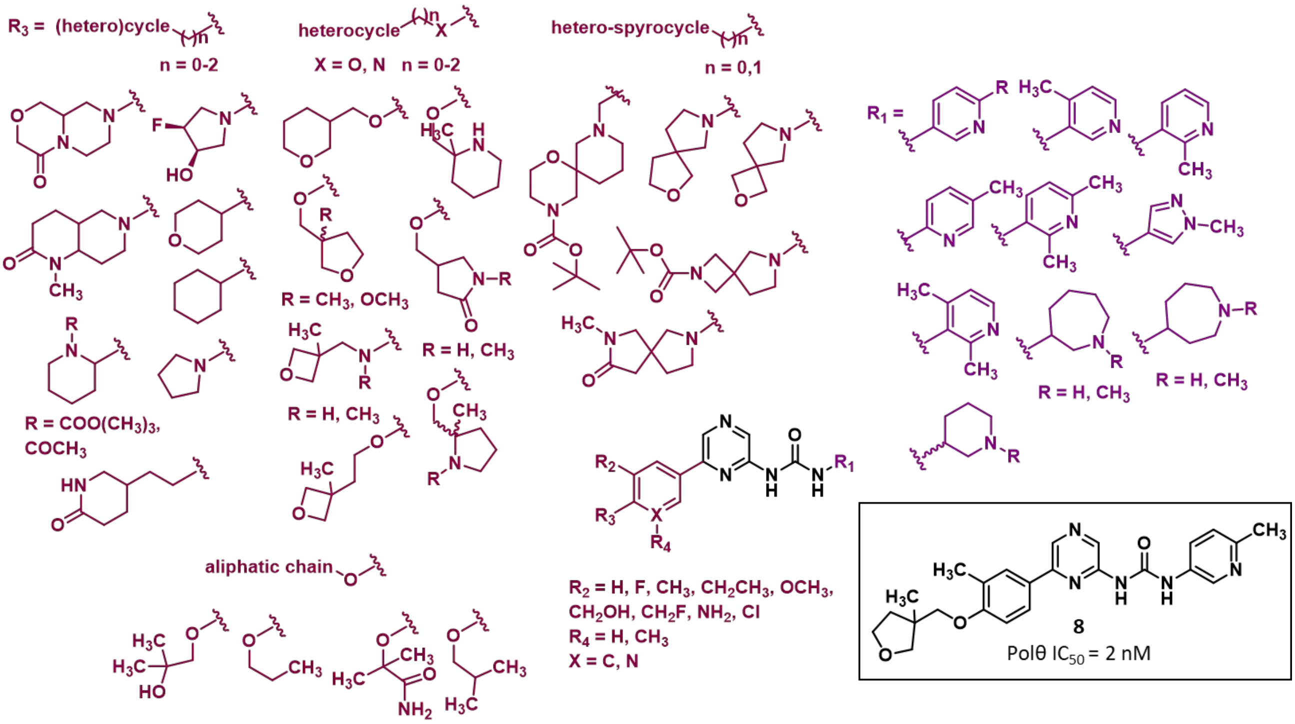

Figure S8. Structure of **heterocyclic substituted urea derivatives** as Polθ-pol inhibitors reported in PA WO2020/030925A1 by Artios.[8] Due to the high number of R_3 substituents, only some representative examples were reported. The IC_{50} value represents the compound concentration that reduces by 50% the Polθ-pol activity as measured by PEA. Most of the compounds showed a 6-methyl pyridine-3-yl ring as aromatic portion linked to the pyrazine core. Among the substituents of the phenyl ring (R_2, R_3 and R_4), R_3 was the most explored by studying a number of different aliphatic groups, of which the morpholine ring was the most frequent, followed by a 3,6-dihydro-2*H*-pyran. Most of compounds showed a methyl group as R_2 substituent, while only few compounds had the R_4 substituent.

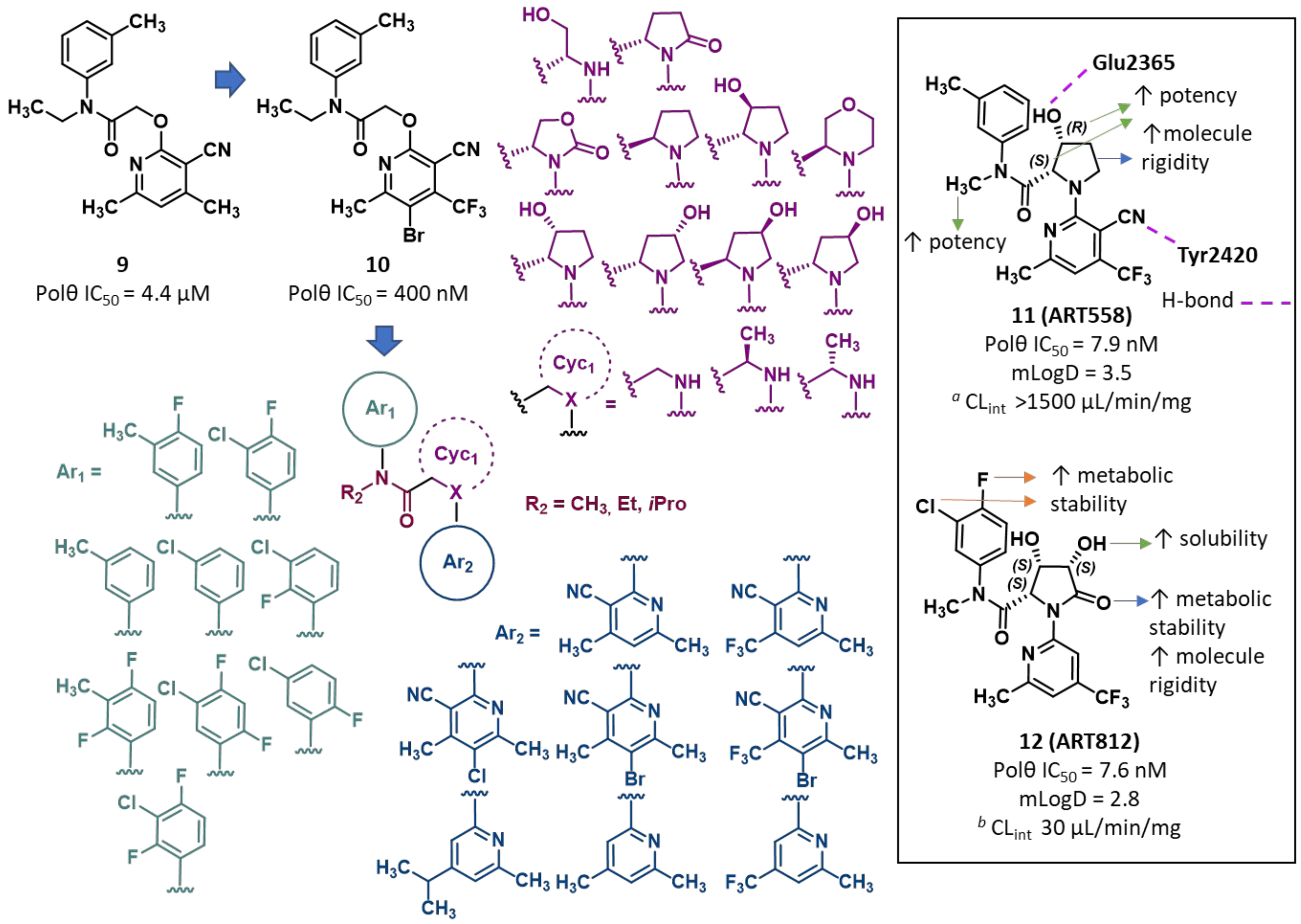

Figure S9. Structure and activity of Polθ-pol inhibitors **ART558**, **ART812** and analogues.[9] The IC_{50} value represents the compound concentration that reduces by 50% the Polθ-pol activity as measured by PEA. The CL_{int} value represents the clearance in [a] mouse and [b] human microsomes. In compound **ART558**, the 2-(*S*)-stereochemistry at the pyrrolidine ring was critical for potency, and, to a lesser extent, also the position of the 3-hydoxyl group and the (*R*)-stereochemistry of the carbon atom bearing this substituent. During the optimization of **ART558**: i) replacement of the proline ring by a lactam prevented oxidation at position 2, thus improving compound metabolic stability, and also increased molecule conformational restriction with removal of the nitrile moiety; ii) the addition of chlorine and/or fluorine atoms on the phenyl ring (Ar_1) further increased microsomal stability, but also the lipophilicity, while a methyl group on the amidic nitrogen (R_2) boosted the potency, and iii) di-hydroxylation of the lactam ring resulted in excellent potency and reduced lipophilicity, as shown by compound **ART812**.

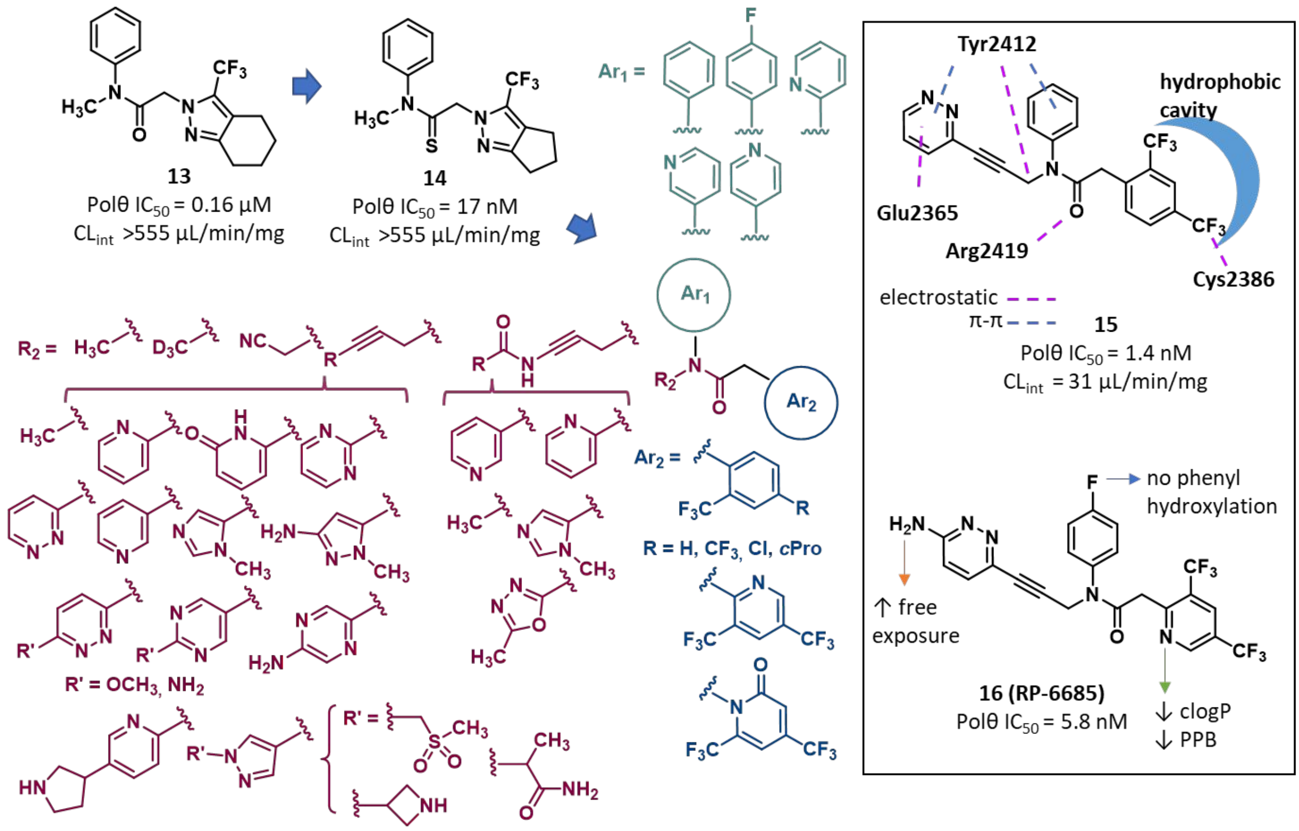

Figure S10. Structure and activity of the Polθ-pol inhibitor **RP-6685** and analogues.[10] The IC_{50} value represents the compound concentration that reduces by 50% the Polθ-pol activity as measured by PEA. The CL_{int} value represents the clearance in mouse microsomes. The *p*-fluorobenzene and 3,5-bis(trifluoromethyl)pyridine were among the best Ar_1 and Ar_2 moieties, respectively.

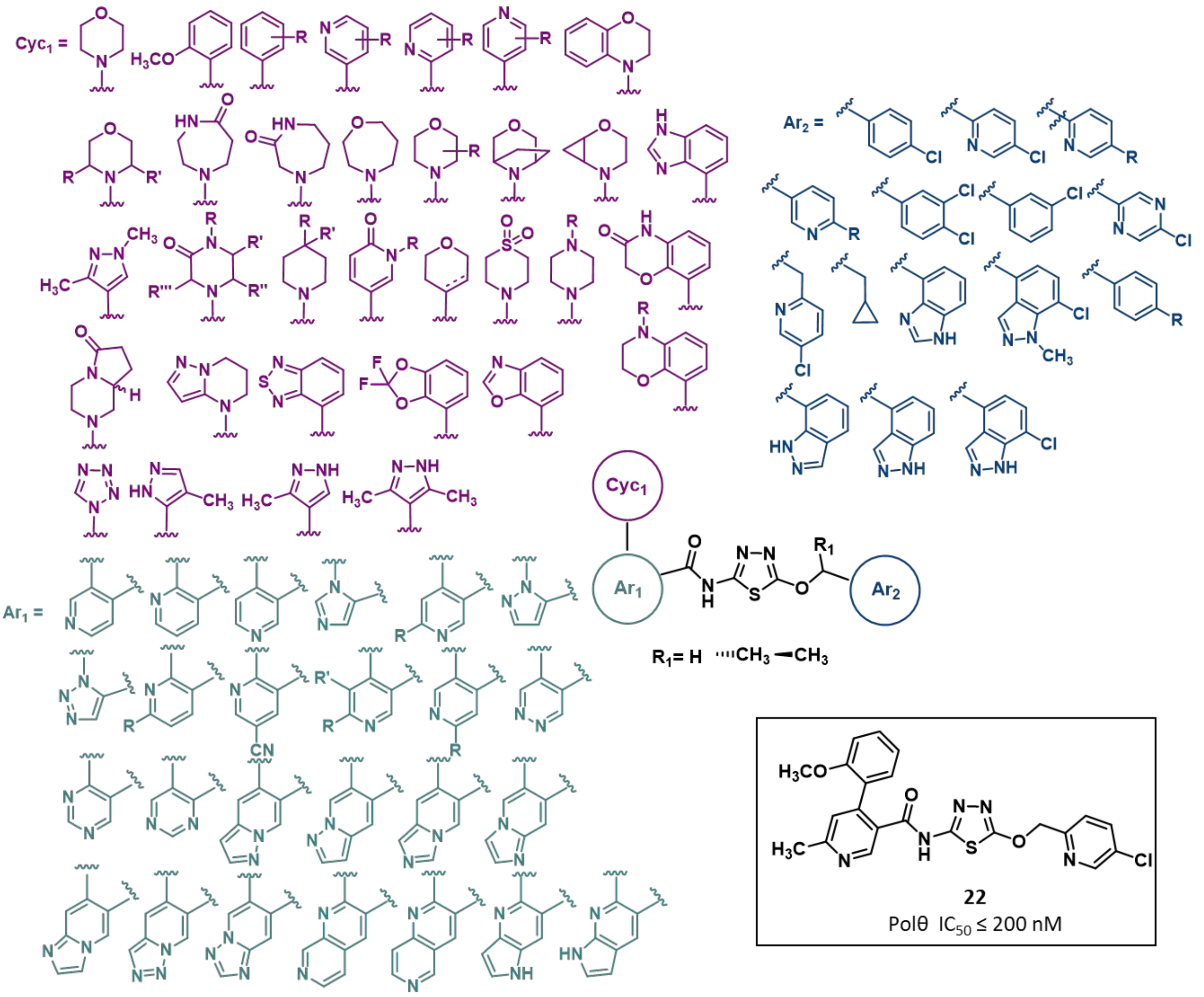

Figure S11. Structure of **thiadiazolyl derivatives** as Polθ-hel inhibitors reported in PA WO2020/243459A1 by Ideaya.[11] Due to the high number of substituents, some of them (R-R''') were omitted. The IC_{50} value represents the compound concentration that reduces by 50% the Polθ-hel activity as measured in the NADH oxidation-coupled enzymatic assay. The most represented Ar_1 moiety was a substituted or an unsubstituted pyridine ring, while a *p*-chlorophenyl and a 5-chloro-2-pyridyl were the most frequent Ar_2 group. A few compounds also displayed a small substituent (R_1) on the methylene of the central portion. Finally, a 2-methoxyphenyl and a morpholine ring were mainly exploited as Cyc_1 portion.

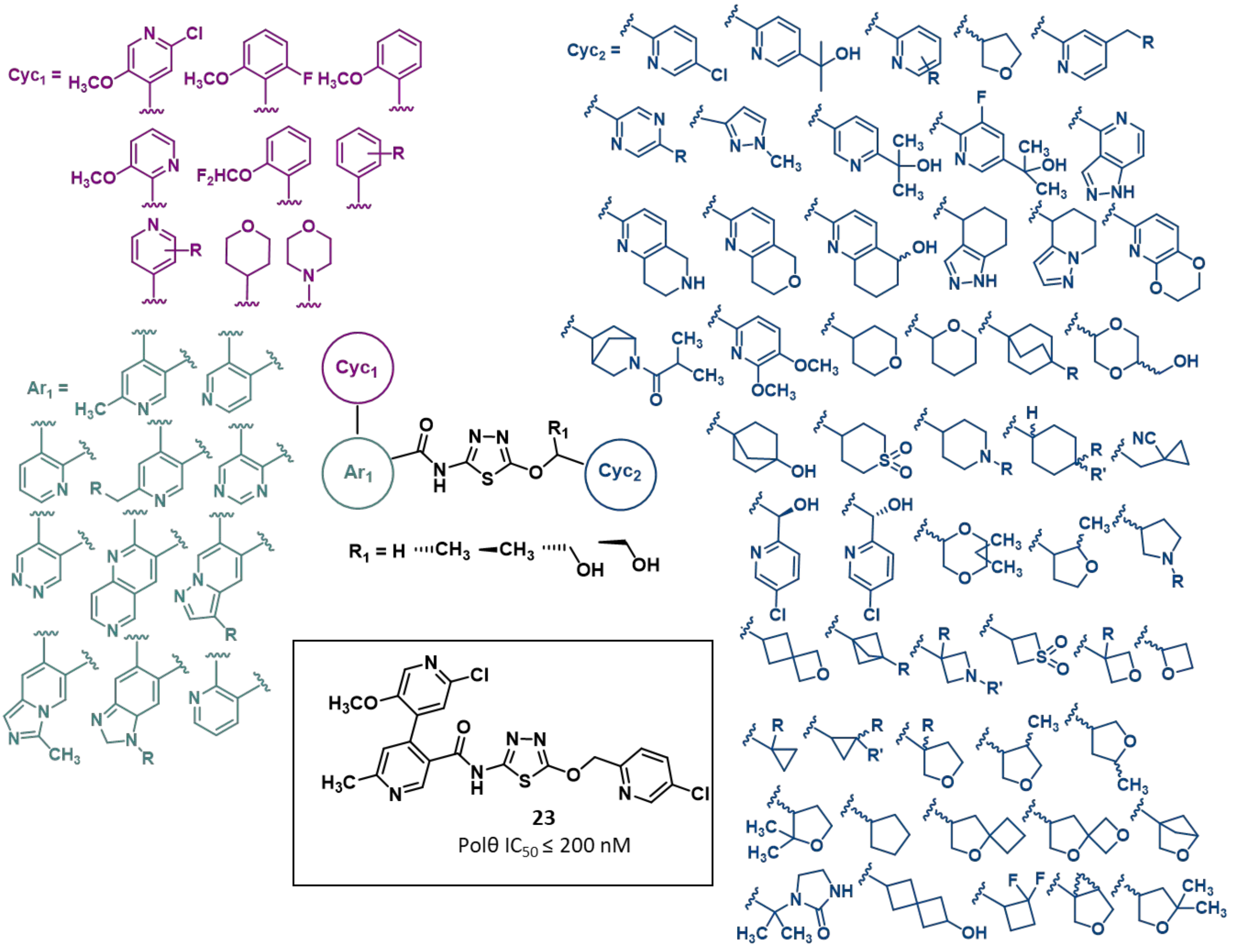

Figure S12. Structure of **thiadiazolyl derivatives** as Polθ-hel inhibitors reported in PA WO2022/118210Al by Ideaya.[12] Due to the high number of substituents, some of them (R and R') were omitted. The IC_{50} value represents the compound concentration that reduces by 50% the Polθ-hel activity as measured by NADH oxidation-coupled enzymatic assay. A 2-methyl pyridine and 2-chloro-6-methoxy-4-pyridyl were mainly exploited as Ar_1 and Cyc_1, respectively. The Cyc_2 was extensively studied by exploring a large number of substituents encompassing both aliphatic and aromatic moieties, although a 5-chloro-2-pyridyl was the most common moiety. Also in this series, a few compounds showed a small R_1 substituent on the methylene unit.

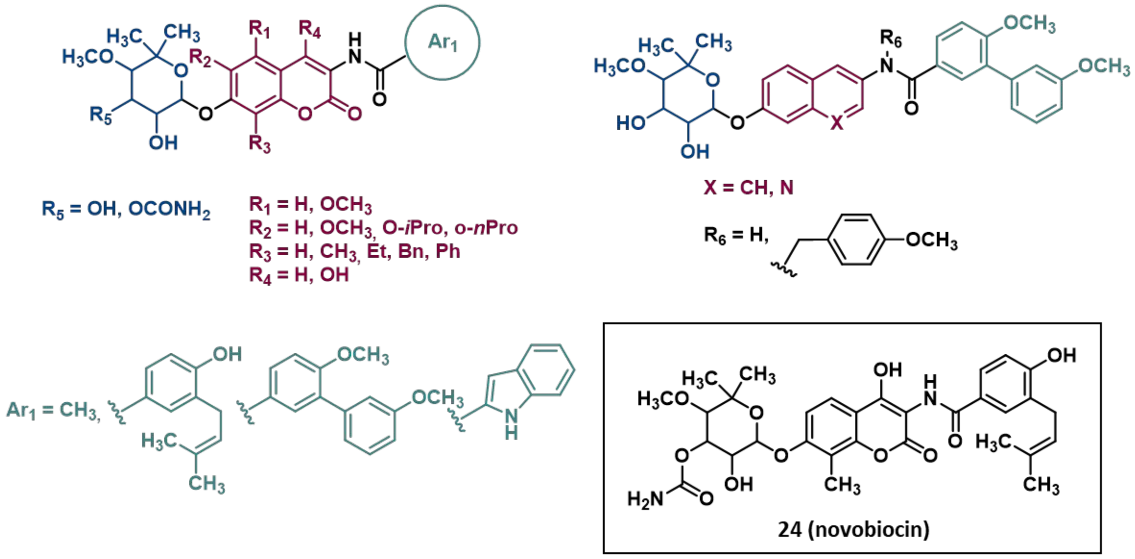

Figure S13. Structure of **2-oxo-2*H*-chromene**, **naphthalene** and **quinoline derivatives** as Polθ-hel inhibitors reported in PA WO2019/079297A1 by Dana-Farber.[13] In many compounds, the 2-oxo-2*H*-chromene scaffold was devoid of the R_1, R_2, and R_4 substituents, while a methyl group was the most common R_3 substituent. The most studied R_5 and Ar_1 substituents were a hydroxyl group and a 3',6-dimethoxy-[1,1'-biphenyl] moiety, respectively.

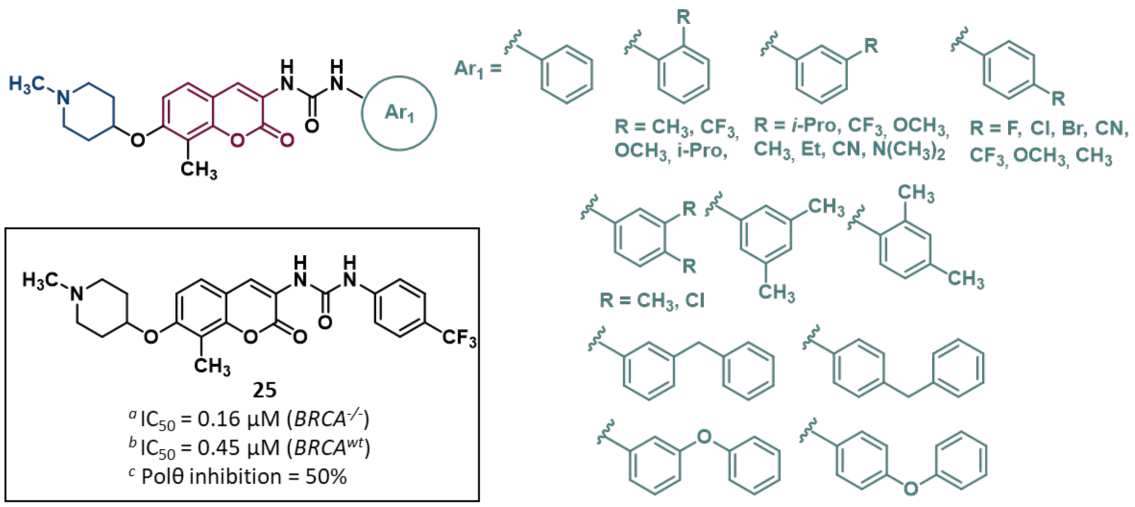

[d] Antiproliferative activity of compound 25

Cell line	MCF7	SKBR3	HCT-116	PC-3	A-549	MDA1986
IC_{50} (µM)	0.46	-	0.8	0.06	0.13	0.9
Cell line	JMAR	B16F10	SKMEL28	MDAMB468LN	MDAMB231	
IC_{50} (µM)	3.6	1.3	6.3	2	0.43	

Figure S14. Structure of **2-oxo-2*H*-chromene derivatives** as Polθ-hel inhibitors reported in PA WO2021/046220Al by Dana-Farber.[14] The IC_{50} value represents the compound concentration that reduces by 50% cell viability (CTG cell viability assay) in [a]*BRCA1*$^{-/-}$ and [b]*BRCA1*^{wt} RPE1 cells. [c]

Percentage of Polθ ATPase activity inhibition determined by ADP-Glo ATPase assay. [d] The IC_{50} value represents the compound concentration that reduces by 50% cell proliferation.

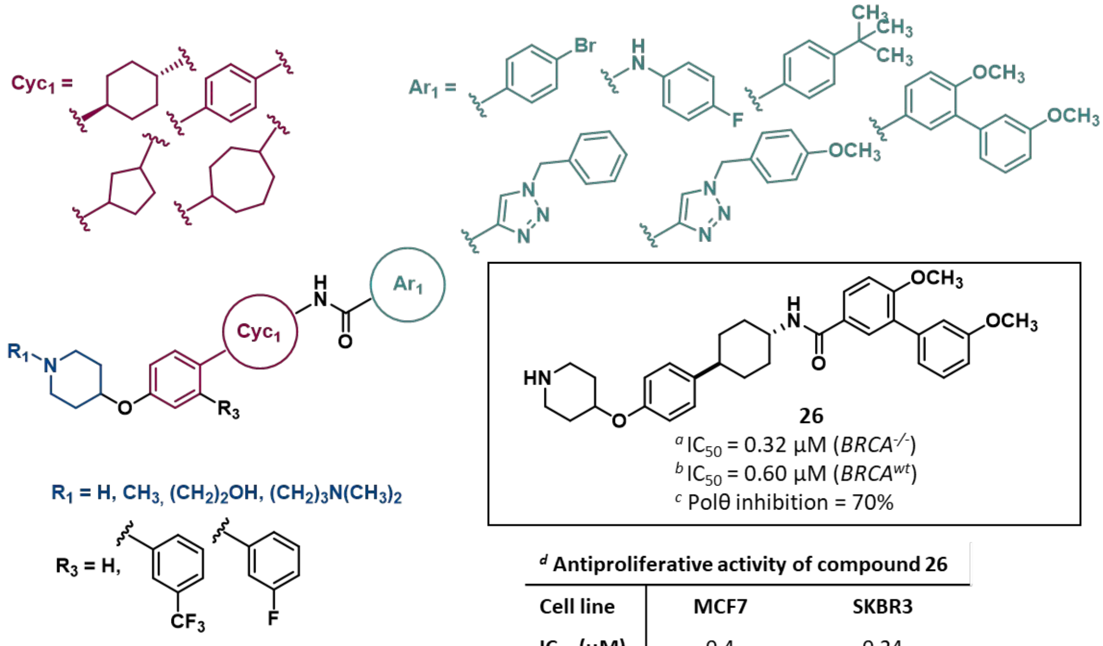

[d] Antiproliferative activity of compound **26**

Cell line	MCF7	SKBR3
IC_{50} (µM)	0.4	0.24

Figure S15. Structure of Polθ-hel inhibitors disclosed in PA WO2021/046178Al by Dana-Farber.[15] [a-d] For the definition of IC_{50} and Polθ inhibition, see **Figure S14** footnote. Ar_1 was a 1,2,3-triazole or phenyl ring, decorated with a small lipophilic substituent or an additional phenyl ring, of which a 3',6-dimethoxy[1,1'-biphenyl] moiety was the most employed. Compounds showing a biphenyl core, also presented an additional phenyl ring (R_3) having a *m*-trifluoromethyl substituent in most of the compounds. All the derivatives showed a piperidin-4-yloxy moiety, in which a methyl group was linked to the nitrogen in most compounds.

Pharmacophore Modelling

To gain insight into the minimal chemical requirements for highly potent Polθ-pol allosteric inhibitors, compounds **17-21** were selected as representative compounds of the five chemical classes A-E, respectively. Each compound was selected as potent compound (IC_{50} < 200 nM) with minimal chemical features. Compounds **17-21** were built using the fragment library tools of Maestro GUI[16] and then submitted to a conformational search using MacroModel.[17] To enhance the conformational sampling, the maximum number of steps was set to 10 000 per molecule. Minimization of conformers was performed using the Polak–Ribiere conjugate gradient method, using a maximum of 500

minimization steps and 0.0005 kJ/(Å mol) as gradient convergence threshold. The energy minimum conformations were used in the development of a common feature pharmacophore model by using Phase.[18,19] For the generation of the pharmacophore model fifty conformers for each ligand were created and minimized. Pharmacophoric models in which at least one training set compound showed a low fitness value (i.e. < 1.8) were discarded. The best-identified hypotheses (AHRR_1, see **Table S1**) was characterized by the following scores: PhaseHypoScore = 1.266; BEDROC score = 1.0; Survival score = 4.44.

Table S1. Features composing the 3D-pharmacophore model developed using Phase (Schrödinger suite) and distances (Dist) between the four pharmacophoric elements.

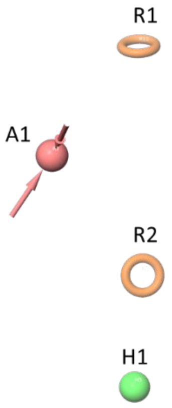

	Dist (Å)
R1-H1	8.92
R1-A1	4.96
R1-R2	6.03
R2-H1	2.90
R2-A1	4.68
A1-H1	6.85

References

(1) WO2020160213 HETEROARYLMETHYLENE DERIVATIVES AS DNA POLYMERASE THETA INHIBITORS.

(2) WO2020160134 ACETAMIDO DERIVATIVES AS DNA POLYMERASE THETA INHIBITORS.

(3) WO2022026548 ACETAMIDO-AMINO AND ACETAMIDO-SULFUR DERIVATIVES AS DNA POLYMERASE THETA INHIBITORS.

(4) WO2022026565 CYCLIZED ACETAMIDO DERIVATIVES AS DNA POLYMERASE THETA INHIBITORS.

(5) WO2021028643 HETEROCYCLIC COMPOUNDS FOR USE IN THE TREATMENT OF CANCER.

(6) WO2021123785 DNA POLYMERASE THETA INHIBITORS.

(7) WO2020030924 THIAZOLEUREAS AS ANTICANCER AGENTS.

(8) WO2020030925 HETEROCYCLIC SUBSTITUTED UREAS, FOR USE AGAINST CANCER.

(9) Stockley, M. L.; Ferdinand, A.; Benedetti, G.; Blencowe, P.; Boyd, S. M.; Calder, M.; Charles, M. D.; Edwardes, L. V.; Ekwuru, T.; Finch, H.; Galbiati, A.; Geo, L.; Grande, D.; Grinkevich, V.; Holliday, N. D.; Krajewski, W. W.; MacDonald, E.; Majithiya, J. B.; McCarron, H.; McWhirter, C. L.; Patel, V.; Pedder, C.; Rajendra, E.; Ranzani, M.; Rigoreau, L. J. M.; Robinson, H. M. R.; Schaedler, T.; Sirina, J.; Smith, G. C. M.; Swarbrick, M. E.; Turnbull, A. P.; Willis, S.; Heald, R. A. Discovery, Characterization, and Structure-Based Optimization of Small-Molecule In Vitro and In Vivo Probes for Human DNA Polymerase Theta. *J. Med. Chem.* **2022**, *65* (20), 13879–13891. https://doi.org/10.1021/ACS.JMEDCHEM.2C01142.

(10) Bubenik, M.; Mader, P.; Mochirian, P.; Vallée, F.; Clark, J.; Truchon, J.-F.; Perryman, A. L.; Pau, V.; Kurinov, I.; Zahn, K. E.; Leclaire, M.-E.; Papp, R.; Mathieu, M.-C.; Hamel, M.; Duffy, N. M.; Godbout, C.; Casas-Selves, M.; Falgueyret, J.-P.; Baruah, P. S.; Nicolas, O.; Stocco, R.; Poirier, H.; Martino, G.; Fortin, A. B.; Roulston, A.; Chefson, A.; Dorich, S.; St-Onge, M.; Patel, P.; Pellerin, C.; Ciblat, S.; Pinter, T.; Barabé, F.; Bakkouri, M. El; Parikh, P.; Gervais, C.; Sfeir, A.; Mamane, Y.; Morris, S. J.; Black, W. C.; Sicheri, F.; Gallant, M. Identification of RP-6685, an Orally Bioavailable Compound That Inhibits the DNA Polymerase Activity of Polθ. *J. Med. Chem.* **2022**, *65* (19), 13198–13215. https://doi.org/10.1021/ACS.JMEDCHEM.2C00998.

(11) WO2020243459 THIADIAZOLYL DERIVATIVES AS DNA POLYMERASE THETA INHIBITORS.

(12) WO2022118210 SUBSTITUTED THIADIAZOLYL DERIVATIVES AS DNA POLYMERASE THETA INHIBITORS.

(13) WO2019079297 COMPOUNDS AND METHODS FOR TREATING CANCER.

(14) WO2021046220 COMPOUNDS AND METHODS FOR TREATING CANCER.

(15) WO2021046178 COMPOUNDS AND METHODS FOR TREATING CANCER.

(16) Schrödinger Release 2019-2: Maestro, Schrödinger, LLC, New York, NY, 2019.

(17) Schrödinger Release 2019-2: MacroModel, Schrödinger, LLC, New York, NY, 2019.

(18) Schrödinger Release 2019-2: Phase, Schrödinger, LLC, New York, NY, 2019.

(19) Dixon, S. L.; Smondyrev, A. M.; Knoll, E. H.; Rao, S. N.; Shaw, D. E.; Friesner, R. A. PHASE: A New Engine for Pharmacophore Perception, 3D QSAR Model Development, and 3D Database Screening. 1. Methodology and Preliminary Results. *J. Comput. Aided Mol. Des.* **2006**, *20*, 647–671.
